# Understanding the Stabilizing Effect of Histidine on mAb Aggregation: A Molecular Dynamics Study

**DOI:** 10.1021/acs.molpharmaceut.2c00453

**Published:** 2022-08-10

**Authors:** Suman Saurabh, Cavan Kalonia, Zongyi Li, Peter Hollowell, Thomas Waigh, Peixun Li, John Webster, John M. Seddon, Jian R. Lu, Fernando Bresme

**Affiliations:** † Department of Chemistry, Molecular Sciences Research Hub Imperial College, London W12 0BZ, United Kingdom; ‡ Dosage Form Design and Development, BioPharmaceutical Development, BioPharmaceuticals R&D, AstraZeneca, Gaithersburg 20878, Maryland, United States; § Biological Physics Group, School of Physics and Astronomy, Faculty of Science and Engineering, Oxford Road, The University of Manchester, Manchester M13 9PL, U.K.; ∥ Photon Science Institute, The University of Manchester, Manchester M13 9PL, U.K.; ⊥ STFC ISIS Facility, Rutherford Appleton Laboratory, Didcot OX11 0QX, U.K.

**Keywords:** Monoclonal Antibodies, Histidine, Molecular Dynamics, Protein Aggregation, Spatial Aggregation Propensity, COE3

## Abstract

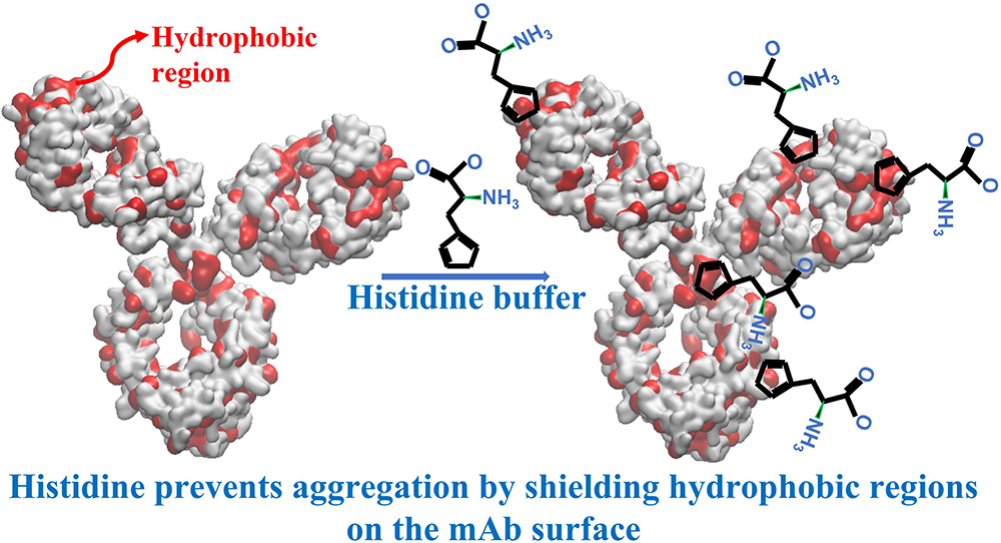

Histidine, a widely used buffer in monoclonal antibody (mAb) formulations, is known to reduce antibody aggregation. While experimental studies suggest a nonelectrostatic, nonstructural (relating to secondary structure preservation) origin of the phenomenon, the underlying microscopic mechanism behind the histidine action is still unknown. Understanding this mechanism will help evaluate and predict the stabilizing effect of this buffer under different experimental conditions and for different mAbs. We have used all-atom molecular dynamics simulations and contact-based free energy calculations to investigate molecular-level interactions between the histidine buffer and mAbs, which lead to the observed stability of therapeutic formulations in the presence of histidine. We reformulate the Spatial Aggregation Propensity index by including the buffer–protein interactions. The buffer adsorption on the protein surface leads to lower exposure of the hydrophobic regions to water. Our analysis indicates that the mechanism behind the stabilizing action of histidine is connected to the shielding of the solvent-exposed hydrophobic regions on the protein surface by the buffer molecules.

## Introduction

Monoclonal antibodies (mAbs) are an important class of therapeutic proteins with applications in cancer, autoimmune, and infectious diseases as well as certain metabolic disorders.^1,2^ Antibody dosage requirements strongly depend on the desired application. Intravenous administration, for instance, can be formulated at low concentrations, while subcutaneous or intramuscular administration typically require concentrated solutions due to volume constraints. High-concentration antibody formulations are often prone to aggregation during manufacturing, storage, and transportation, which motivates us to develop methods to predict aggregation in the pharmaceutical industry. Particularly, tools that provide microscopic insights into the mAb’s solvation structure and conformation in solution, as well as mAb-buffer interactions, might contribute to devising strategies to enhance the stability of mAb suspensions during long-term storage. Changes in the pH of the solution influence the protein charge and could lead to unstable protein formulations. Hence, protein formulations rely on buffers such as histidine, acetate, citrate, aspartate, phosphate, or tris to maintain the solution pH.^3−7^ Histidine is one of the most widely used amino acid buffers, as the transition between the neutral and the +1 charged state takes place at pH = 6,^8^ very close to the pH at which most mAbs display optimal stability. Histidine is also known to effectively stabilize mAbs against aggregation. Kalonia et al.^9^ performed solubility measurements of IgG1 mAb, showing that the histidine buffer provided better stability against aggregation than citrate, at pH values between 4.5 and 6.5. They also found, using static light-scattering measurements, that the mAb–mAb interaction in the presence of histidine is repulsive. Size exclusion chromatography experiments demonstrated that histidine impedes monomer loss from solution even at elevated temperatures of 40 and 57 °C, implying that histidine is capable of stabilizing suspensions of both native and non-native mAbs.

Previous studies showed that the stabilizing capacity of some excipients, like sucrose, correlates with their ability to preserve the secondary structure of mAbs.^10^ For histidine, however, the stabilizing capacity seems to not correlate with secondary structure preservation.^10^ Fourier transform infrared (FTIR) experiments demonstrated that the secondary structure of the dried antibody ABX-IL8 was similar for formulations containing 4 or 6 mM histidine (69% β-sheet), while the stability of the antibody against aggregation in the lyophilized state varied significantly with the amount of histidine. Furthermore, increasing the concentration of histidine in solution inhibited aggregation to a larger extent and reduced the viscosity of the solution.^10^ These experiments suggest that the stabilizing impact of histidine on mAb formulations does not depend solely on its ability to preserve the mAb structure, and other mechanisms, possibly connected to the modification of the surface chemistry of the protein, charge screening, modification of surfaces encountered during storage, and the interaction of mAb with these surfaces must be taken into account.^11^

Experimental studies of Histidine/IgG4-mAb interaction^12^ using Dynamic Light Scattering experiments highlighted the importance of electrostatic interactions. Significant changes in the hydrodynamic radius of the antibody, with increasing histidine concentration (from ∼5 nm at 1 mM histidine to ∼6.5 nm at 20 mM), were observed at a pH of 5.8. Interestingly, the correlation between the hydrodynamic radius and the amount of histidine was not linear, and further increase of the amount of histidine in solution led to a reduction in the hydrodynamic radius. In contrast, at neutral pH, the hydrodynamic radius featured negligible changes with histidine concentration. The positive charge of the protein and the fraction of charged buffer histidines decreases with increasing pH. For an increase of pH from 5.8 to 7, for instance, the fraction of charged buffer histidines decreases from 60% to 8%. This would lead to a weaker electrostatic interaction between the protein and the buffer resulting in a negligible dependence of the protein size on histidine concentration. However, if the change in protein size is due purely to electrostatic effects, one would expect a change in the ionic strength of the solution (e.g., by adding NaCl) to have a measurable impact. On the contrary, experiments indicate that adding NaCl at constant histidine concentration does not influence the hydrodynamic radius.^12^ Overall, the experimental studies point toward a more particular role of histidine, possibly linked to specific histidine-antibody interactions. However, the microscopic mechanism behind the histidine-mediated stabilization of mAb solutions is still unknown. We investigate this mechanism in this work, using all-atom Molecular Dynamics (MD) simulations. Hence, our work significantly aligns with the aspirations of the community to identify low-cost approaches that can assist drug development, as reflected in recent works.^13−16^ Here we quantify the interactions between mAb COE3 (and its Fab and Fc fragments) and histidine in aqueous solution and identify the preferred sites for histidine adsorption on COE3. We introduce the BSAP index, which is an extension of the Structural Aggregation Propensity (SAP) metric introduced by Chennamsetty et al.^17^ The BSAP index incorporates changes in the effective hydrophobicity of the antibody, which are associated with the adsorption of the buffer on the antibody’s surface.

Finally, we note that substantial work has been performed linking aggregation to potential immunogenicity.^18,19^ There have also been multiple studies linking citrate to promoting increased protein–protein interactions and aggregation in monoclonal antibody formulations.^9,20^ Here, we demonstrate that histidine blocks the hydrophobic regions of the protein. This result supports the experimental observation on the impact of histidine reducing protein–protein interaction, which potentially inhibits aggregation, reducing the risk of immunogenic responses associated with product degradation. Therefore, the selection of formulation buffer (e.g., histidine vs citrate) could impact product quality, affecting immunogenicity.

## Materials and Methods

### Molecular Dynamics of Protein and Histidine Solutions

#### Generating Initial Models of the Fab/Fc Fragments

The sequence of the Fc fragment of COE3 is identical to that of the Fc fragment of the human IGG B12 (pdb id: 1HZH(21)), while for the Fab fragments, the sequence similarity is 73%. The initial model of the Fc fragment was obtained by deleting the two Fab fragments from the 1HZH structure. The Fc fragment consists of two protein chains that are sections of the antibody’s two heavy chains. The six disulfide bonds in the fragment (two interchain bonds in the region corresponding to the COE3 hinge and four intrachain bonds, two in each chain) were connected. The initial model of the Fab fragment was obtained from the work by Singh et al.^22^ The Fab fragment consists of two protein chains: the light chain and a part of the heavy chain. The Fab structure has five disulfide bonds, one interchain and four intrachain. The structure of the Fab and Fc fragments and the position of the disulfide bonds are shown in Figure S1A,B, respectively, in the Supporting Information.

#### Setting the Protein Charge

The simulations were performed at pH = 6, a pH within the range of 4–6 commonly employed in monoclonal antibody formulations.^23^ We calculated the protonation state of the titrable residues of the proteins at this pH using the propKa3.0 software.^24^ At pH = 6, Fab has a net charge (+14e) twice as large as that of the Fc fragment (+7e). The pH has a significant impact on the charge of the fragment. The net charge of Fab decreases by 3 units (*q* = +11e) upon increasing the pH from 6 to 7. Lower pH results in the protonation of a GLU residue and two surface-exposed HIS residues. The net charge of the Fc fragment at pH 7 was found to be +2e, which increased to +7e at pH 6, showing a high sensitivity of the charge to the acidic conditions. These changes are driven by the modification of the protonation state of 5 of the HIS residues on the Fc surface. The position of the charged HIS and neutral GLU residues for the Fab and Fc fragments are shown in Figure S1A,B of the Supporting Information.

#### Histidine Buffer Protonation States

The simulations were performed at an l-histidine (L-HIS) buffer concentration of 20 mM, which is a typical concentration used in mAb formulations.

**Figure 1:**
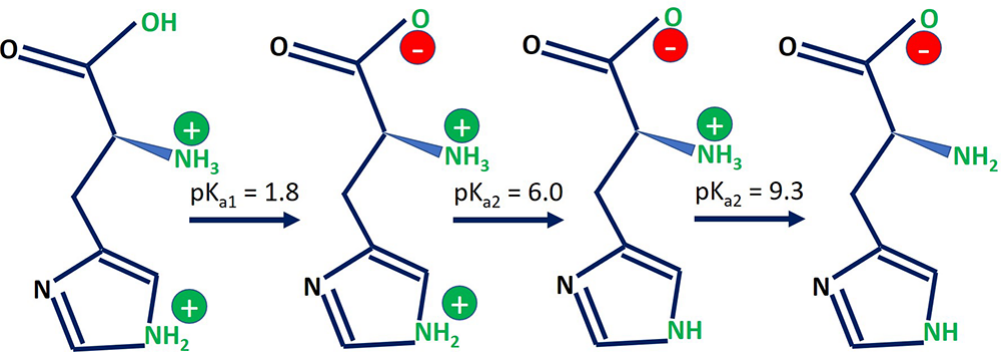
p*K*_a_ values for the histidine molecule. At pH = 6, histidine has an equal probability of being in the +1 charged state or the neutral state.

**Figure 2:**
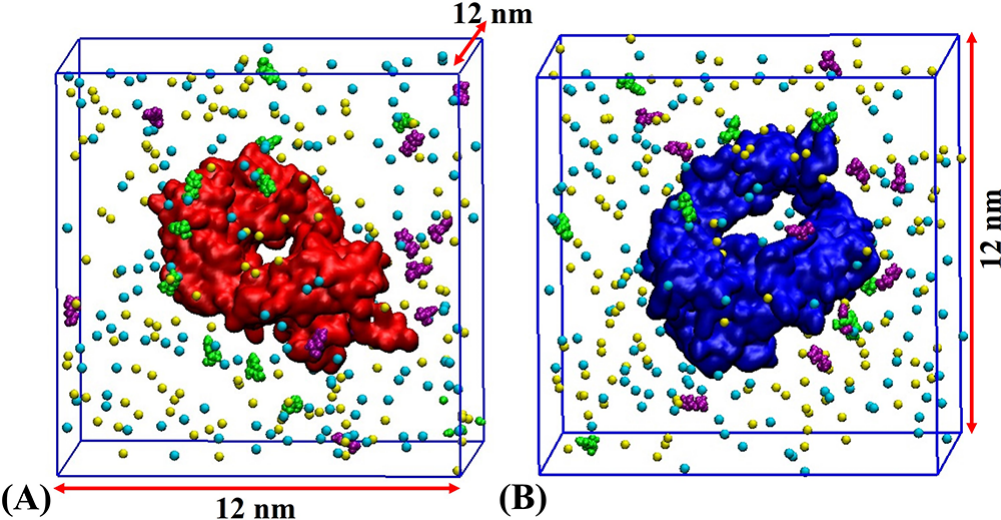
Snapshots of the initial systems for (A) Fab and (B) Fc domains in a solution containing 20 mM of histidine. The Fab fragment is shown in red, the Fc fragment is shown in blue, Na^+^ in cyan, and Cl^–^ in yellow. HIS^0^ and HIS^+^ are shown in green and purple, respectively. Water molecules are not shown for clarity.

At pH = 6, histidine transitions from a charged to a neutral form (see Figure 1). The fraction of charged histidine residues, *f*_charged_, was calculated using the Henderson–Hasselbalch equation:^25,26^
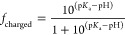
1At pH = 6, eq 1 predicts half of the buffer histidine molecules to have a net positive charge. For our system size for the Fab/Fc simulations, a buffer concentration of 20 mM required the addition of 20 histidine molecules. The simulated buffer thus consisted of 10 positively charged (HIS^+^) and 10 neutral (HIS^0^) histidines. The histidines were added randomly in the periodic simulation box containing the protein at the center (see Figure 2). At pH = 6 the terminal amine and carboxyl groups of histidine are charged (+1 and −1, respectively, see Figure 1) resulting in 1:1 zwitterionic/cationic histidines dispersed in the buffer solution.

### Simulation Protocol

The histidine-protein system was solvated in water, and the interactions between the water molecules were modeled using the mTIP3P^27^ water model. This model has been used to parametrize the CHARMM27^28^ force field, and it is identical in structure and parameters to the original TIP3P model, with the exception of having a weak Lennard-Jones interaction for the hydrogens. Following the solvation process, we added Cl^–^ ions (24 for the Fab and 17 for the Fc fragment) to neutralize the charges of the buffer and the protein. In addition, 148 Na^+^ and an equal number of Cl^–^ ions were added for a salt concentration 150 mM. Further, to quantify the impact of the buffer on the structural properties of the proteins, we also performed simulations at pH = 6, without any buffer. We list in Table 1 the details of all the simulations performed in this work and the corresponding system sizes. All the simulations reported in this work were performed using the GROMACS(2018.2) software^29,30^ package. The systems were first minimized using the steepest descent method with all the protein atoms held fixed with harmonic restraints (force constant, 1000 kJ/(mol nm^2^)) to their initial positions to remove bad contacts between the water molecules, ions, and atoms belonging to the protein. Following minimization, the systems were pre-equilibrated for 1 ns in the NVT ensemble at a temperature of 300 K, again keeping the solute atoms restrained at their respective positions. The systems were then subjected to a 1 ns long unrestrained equilibration, in the NPT ensemble, at a constant temperature of 300 K and pressure of 1 bar. Following the equilibration, 200 ns long production runs were performed in the NPT ensemble. In all our simulations the canonical v-rescale thermostat^31^ was used for temperature control with a temperature coupling constant of 0.5 ps. During equilibration we used the Berendsen barostat,^32^ with a pressure coupling constant of 0.5 ps, while the Parrinello–Rahman barostat^33^ (coupling constant of 2.0 ps) was used during production. The Particle Mesh Ewald^34^ method was used for evaluating the electrostatic interactions. We employed a cutoff of 1 nm for the dispersion interactions. Long-range pressure corrections were not included. A simulation time step of 2 fs was employed, and the bonds involving hydrogens were held rigid using the LINCS algorithm.^35^

**Table 1:** Summary of the Systems Simulated in This Work^a^

S.no	system details	*N*_H_2_O_	*N*_HIS_	*N*_ATOM_	time (ns)
1.	Fab with L-HIS	54048–55	20	169464–85	200 × 3
2.	Fc with L-HIS	53917–25	20	169415–39	200 × 3
3.	Fab with no buffer	54222	0	169566	100 × 3
4.	Fc with no buffer	54089	0	169511	100 × 3
5.	COE3 with L-HIS	253566–619	20	784435–597	100 × 4
6.	COE3 with no buffer	254439	0	784893–5074	100 × 3

a The simulations were performed with the CHARMM27 force field for the ions and amino acids. See Materials and Methods for details on the charges of titrable amino acids of the proteins and the buffer histidine. *N*_H_2_O_ and *N*_HIS_ represent the number of water and histidine molecules, and *N*_ATOM_ indicates the total number of atoms for each system. For the Fab and Fc systems with buffer, the initial position of buffer molecules were different for each of the three independent simulations leading to a range of system sizes (169464-169485 for Fab and 169415-169439 for Fc). For the COE3 systems, the range of system sizes originates from the initial buffer positions and the different antibody starting conformations employed for each independent run. Time indicates the simulation time for production and calculation of time averages.

### Simulation of the Antibody COE3

**Figure 3:**
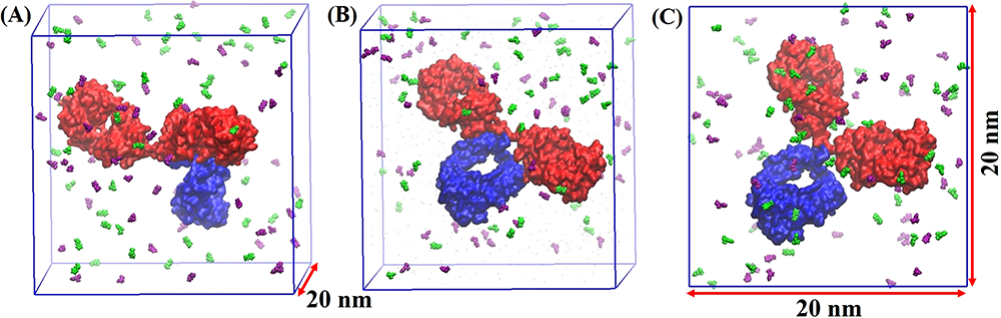
Snapshots of the initial systems for the four independent runs of the full antibody, COE3. Two independent simulations were performed starting from the conformation shown in (C). HIS^0^ and HIS^+^ are shown in green and purple, respectively. Water and ions are not shown as spheres for clarity.

Three different initial antibody structures were built by combining one Fc fragment and two Fab fragments with different relative orientations leading to three different antibody conformations. The first conformation was generated by aligning the Fab and Fc domains with those of the mAb crystal structure with PDB id:1HZH^21^ (see Figure 3A). The other two conformations were planar with both the Fab domains either in or out of contact with the Fc domain (see Figure 3B,C, respectively).

The 16 disulfide bonds present in the antibody (four in the Fc domain, five in each of the Fab domains, and two in the hinge region) were connected. The charges of different titratable residues were obtained using the propKa3.0 software. The total charge of the antibody was fixed to *q* = +36e. The total charge of the antibody is one more than the sum of charges used in the simulation of the individual fragments because our propKa analysis predicts an extra GLU residue at the interface of the Fab and Fc surfaces of COE3 to be neutral (see Figure S1 of Supporting Information). The structures with appropriate charges were enclosed in a cubic box of length 20 nm. 51 positively charged (HIS^+^) and an equal number of neutral (HIS^0^) histidine residues were added randomly to the box containing the antibody, constituting the buffer at a concentration of 20 mM. The systems were solvated in mTIP3P water molecules. 87 Cl^–^ ions were added to neutralize the mAb and HIS^+^ charges. Further, 723 Na^+^ and an equal number of Cl^–^ ions were added to attain a salt concentration of ∼150 mM. Information on the system sizes is provided in Table 1. To speed up the conformational sampling of the proteins in water, we performed simulations with masses for the hydrogen and oxygen atoms in water scaled by a factor of 1/10th of the original mass. This mass change does not impact the configurational properties since for the classical Hamiltonian employed here
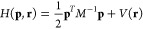
2where *M*^−1^ is the inverse of the mass tensor, the momentum (**p**) and potential contributions (*V*(**r**)) are separable, and the position-dependent properties, *A*, only depend on the latter.
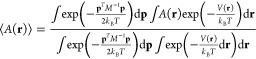
3This approach has been successfully used by Lin et al.^36^ to enhance the conformational sampling of peptides in solution. To evaluate the effect of using a scaled water mass, we performed simulations of 1378 water molecules enclosed in a periodic box of volume *V* = (3.576)^3^ nm^3^, with original and scaled water masses, in the NVT ensemble, at a temperature of 300 K. The viscosities were obtained using the Green–Kubo relation
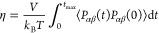
4and averaged over the three off-diagonal components of the pressure tensor, *P*_αβ_, {α, β} = {*x*,*y*}, {*x*,*z*}, {*y*,*z*}. The calculation was performed over trajectories spanning 20 ns, integrating the correlation function up to *t*_max_ = 5 ps. The reduction in the mass results in a significant decrease in the viscosity of water, from η_o_ = 0.348 ± 0.005 mPa s to η_s_ = 0.129 ± 0.005 mPa s, for the original and scaled masses, respectively. This reduction in viscosity results in a decrease of the characteristic time for diffusion of the solutes by a factor of η_o_/η_s_ ≈ 2.7, which is significant for the protein sizes considered here.

### Histidine Adsorption on the Protein

We identified the shortest of all atomic-pair distances, *d*_min_, between each buffer histidine molecule and the protein. *d*_min_ defines the separation between a histidine molecule and the protein surface. A histidine molecule was deemed to be adsorbed on the protein if *d*_min_ ≤ 0.4 nm. The cutoff was set such that both hydrogen bonds (cutoff acceptor-hydrogen distance of ∼0.25 nm) and salt-bridges (cutoff distance of ∼0.4 nm) are included.^37^

**Figure 4:**
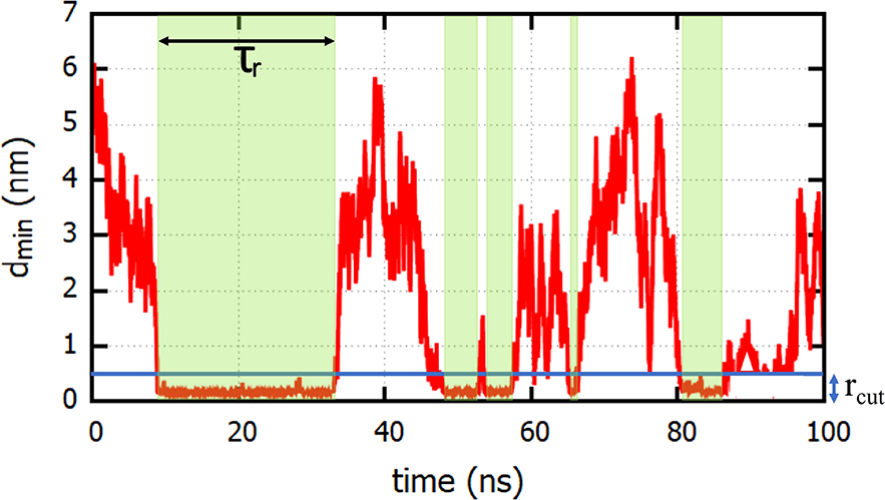
Time dependence of the minimum distance, *d*_min_, of a buffer histidine molecule from the Fc surface. The regions of the trajectory where *d*_min_ is below *r*_cut_ = 0.4 nm correspond to adsorption events (shaded in green), while the rest of the trajectory corresponds to free diffusion of histidine in solution. The length of an adsorption event τ_r_ defines a residence time.

The time series of *d*_min_ was calculated for each buffer histidine molecule. We show in Figure 4 the variation of *d*_min_ with time for a single histidine molecule. The trajectory can be decomposed into a series of intervals: time regions where the **d**_min_ for a buffer histidine molecule either lies within or beyond a distance of *r*_cut_ from the protein surface. The stretch of time for which *d*_min_ ≤ *r*_cut_ corresponds to a *residence event* and the time interval is called the *residence time* (τ_r_).

The time dependence of *d*_min_ for all buffer histidines was used to study the adsorption kinetics by calculating the survival probability *S*(*t*),
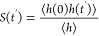
5Here, *h*(0) = 1 if a histidine-protein contact is present at time *t* = 0 and 0 otherwise, while *h*(*t*′) is 1 if a contact, present at *t* = 0, is still intact at time *t* = *t*′. If reattachment takes place due to diffusion of a given histidine back from the solution, we consider this event as a new adsorption event. *S*(*t*) can thus be defined as the probability that a buffer-protein contact that exists at time 0 continues to exist at least up to time *t*. The average residence time of histidine on the protein surface (⟨τ_r_⟩) is defined as the average of all τ_r_ (see Figure 4 for the definition of τ_r_) values for the residence events observed for all buffer molecules over three independent simulations.

*S*(*t*) and (⟨τ_r_⟩) were calculated separately for both HIS^+^ and HIS^0^. As these parameters depend on the strength of a buffer-protein interaction, a comparison provides information on the relative affinities of different buffer histidine charged states toward the protein surface.

## Results and Discussion

### Structure of Fc, Fab, and COE3 in Solution and the Effect of Histidine

**Figure 5:**
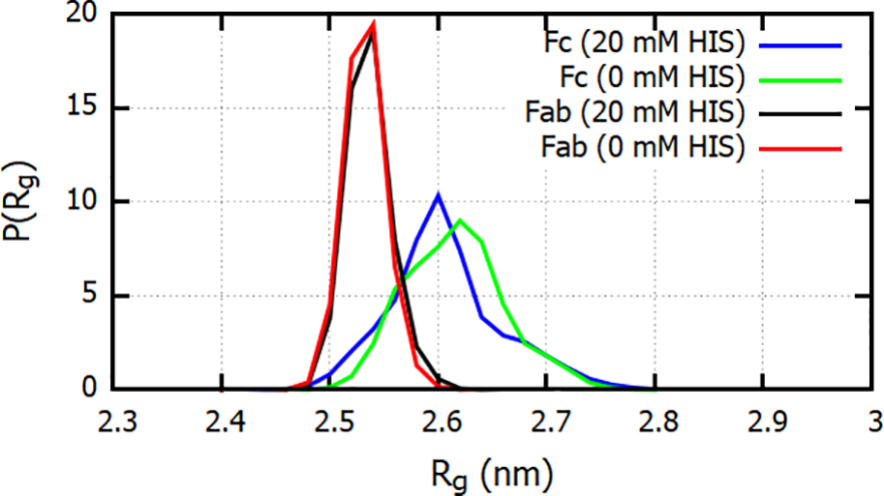
Normalized probability distribution of the radius of gyration of the Fab and Fc fragments at histidine buffer concentrations of 20 and 0 mM. The distributions shown are calculated by averaging over the distributions from three independent MD simulations.

Previous experimental studies showed that histidine might interact with the protein surface, leading to structural changes in the protein.^10,12^ To quantify the degree of structural changes associated with histidine adsorption on the proteins, we computed the probability distribution of the radius of gyration (*R*_g_) of the Fc and Fab fragments, in the presence and absence of histidine. Figure 5 shows the distributions of *R*_g_ for the Fab and Fc fragments, averaged over three independent simulations, each spanning 200 ns. The distributions show almost no change for the Fab fragment and a negligibly small change in the case of the Fc fragment. The averages computed over the three runs, in the presence of histidine, are 2.53 ± 0.03 nm for the Fab fragment and 2.6 ± 0.09 nm for the Fc, which are identical, within statistical uncertainty, to the radii of gyration obtained in the absence of histidine: 2.52 ± 0.02 and 2.6 ± 0.05 nm for Fab and Fc, respectively.

We obtain a broader distribution for the Fc fragment compared to Fab, suggesting that the Fc has a much larger intrinsic flexibility. The width of the distributions for 0 and 20 mM histidine are very similar, indicating a lack of significant correlation between the fluctuations in the protein structure and the presence of the buffer. The time series of *R*_g_ used to calculate the distributions are shown in Figure S2 of the Supporting Information.

**Figure 6:**
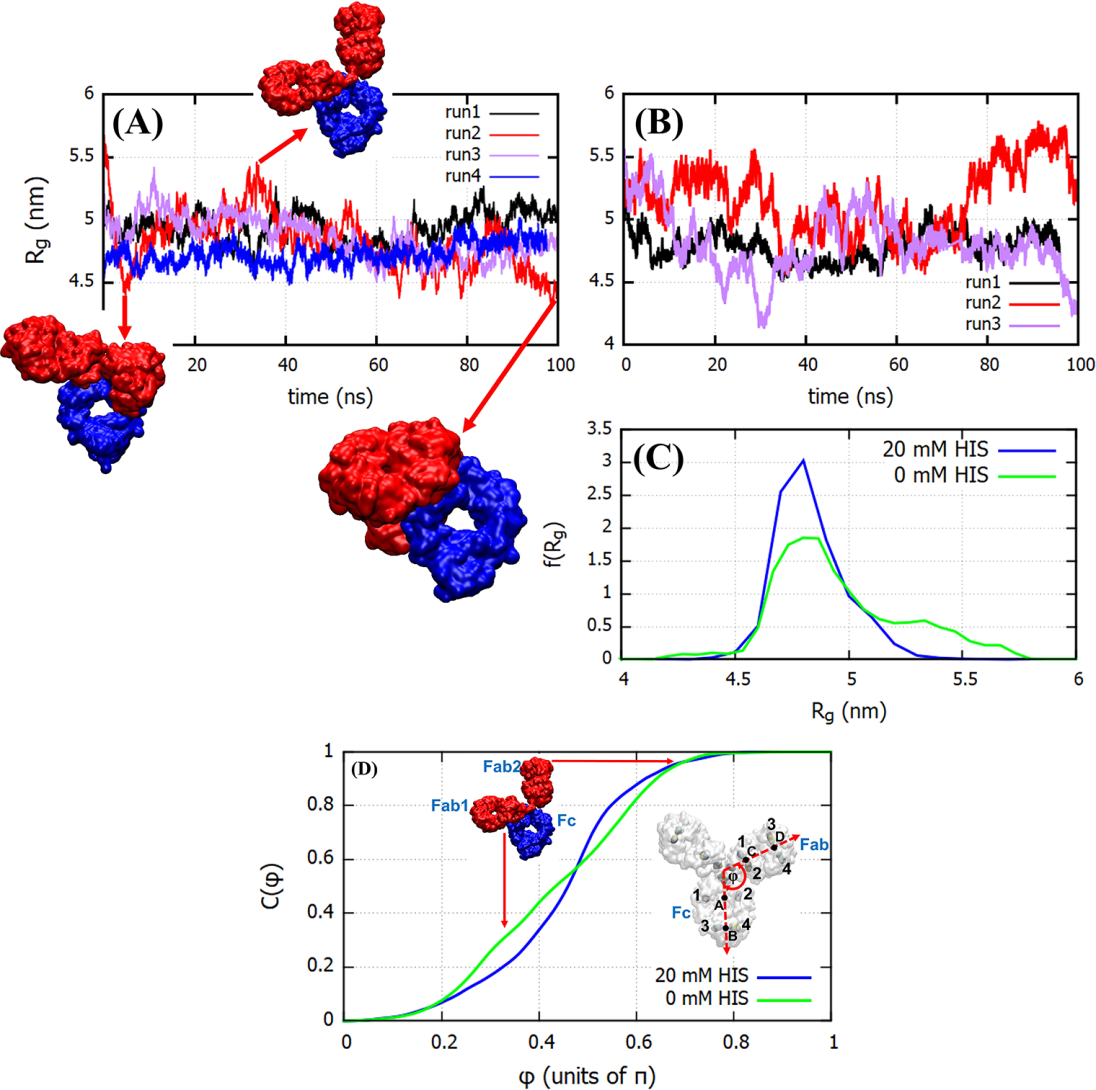
Radius of gyration for the antibody COE3 as a function of time for (A) the four independent MD simulations performed at a buffer concentration of 20 mM and (B) the three independent simulations performed at 0 mM buffer. (C) The distribution of *R*_g_ for 20 and 0 mM buffer. (D) The cumulative distribution of the Fab–Fc angle (φ), defined in the inset, obtained from simulations at 20 and 0 mM buffer. The details shown in the inset in (D) are explained in the main text.

We performed a similar analysis for COE3 using four independent 100 ns trajectories (see Figure 6A). The *R*_g_ of the mAb features significant fluctuations with values ranging from 4.4 to 5.5 nm. A similar range of values for *R*_g_ has been reported in other studies. Clark et al.^38^ investigated, using Monte Carlo simulations, the conformations of an IgG2 antibody. They reported values of *R*_g_ in the range of 3.9–5.5 nm. Recently Tomar et al.^39^ investigated the impact of thermal stress on the flexibility of the IgG_1_κ b12 monoclonal antibody using 100 ns long molecular dynamics simulations. They demonstrated that the antibody is highly flexible and that, under thermal stress, it adopts a more globular shape with a concomitant decrease in the radius of gyration and solvent-accessible surface area. The radius of gyration reported for the b12 mAb is ∼4.9 nm, similar to the value obtained here. Our solvent-accessible surface area (SASA), 673.0 ± 8 nm^2^ is also similar to the values reported in ref (39). The fluctuations in *R*_g_ (see Figure 6) arise from the inherent flexibility of the antibodies, particularly near the hinge region, as demonstrated by the snapshots shown in Figure 6, which depicts a large variation in the relative orientation of the Fab and Fc domains of the antibody with time. The high flexibility in COE3 is consistent with previous results for the b12 mAb.^39^ The *R*_g_ in the presence of histidines (see Figure 6) features significantly smaller fluctuations. This is clearly observed both from the *R*_g_ versus time plots (Figure 6A,B) and the corresponding probability distributions (Figure 6C). We conclude that the histidines inhibit the conformational flexibility of the mAb (cf. the *R*_g_ distributions in Figure 6C). As the conformational flexibility of the antibody arises from its flexible hinge, we infer that the histidines might induce rigidity in the antibody structure through their interaction with the hinge, which ultimately leads to its stiffening. The effect of stiffening of the hinge should be reflected in the parameters defining antibody conformation. In Figure 6D we show the cumulative distribution of the Fab–Fc angle. Vectors were defined using the four pairs of intrachain disulfide bonds in the Fab and Fc domains (see inset of Figure 6D). The angle between the vectors joining the center of mass of cystines forming the disulfide bonds 1 and 2 (point A on Fc and C on Fab) to that of the cystines involved in bonds 3 and 4 (point B on Fc and D on Fab) was defined as the Fab–Fc angle φ. From the distributions shown in Figure 6D we infer that, in the presence of the buffer, the angles in the range of 0.2π < φ < 0.5π are less probable. The smaller values of φ correspond to COE3 conformations with Fab and Fc domains in contact, and the hinge strongly bent (see the position of Fab1 in the inset). A lower sampling of such conformations in the presence of buffer implies that the buffer restricts the hinge flexibility. We also note that the distribution *C*(φ) in the absence of histidine is rather uniform in 0.2π < φ < 0.7π, indicated by the uniform slope of the cumulative distribution in this range. This is consistent with a higher flexibility of the mAb hinge in the absence of histidine. Advancing the discussion below, we will later show that histidine molecules feature significant interactions with the hinge region of the antibody (see Figure 11). Evidence of stiffening of the hinge in the presence of histidine has been observed in experiments. Salinas et al.^40^ showed that the process of denaturant-induced unfolding of mAb involves much lower cooperativity between its three domains, in the presence of histidine buffer. The lack of cooperativity is connected to a lower degree of interfragment interaction. As the prevalence of interfragment interactions depends on hinge flexibility (a flexible hinge allows for the fragments to approach each other closely), the experiments show that the presence of histidine results in a stiffening of the hinge. It has also been seen that histidine reduces the rate of fragmentation of the mAb.^40^ Fragmentation occurs via hydrolysis and initiates in the flexible regions of the antibody. The reduced fragmentation rate of the mAb in histidine buffer is consistent with strong buffer-hinge interactions and the resulting loss of flexibility of the hinge region.

**Figure 7:**
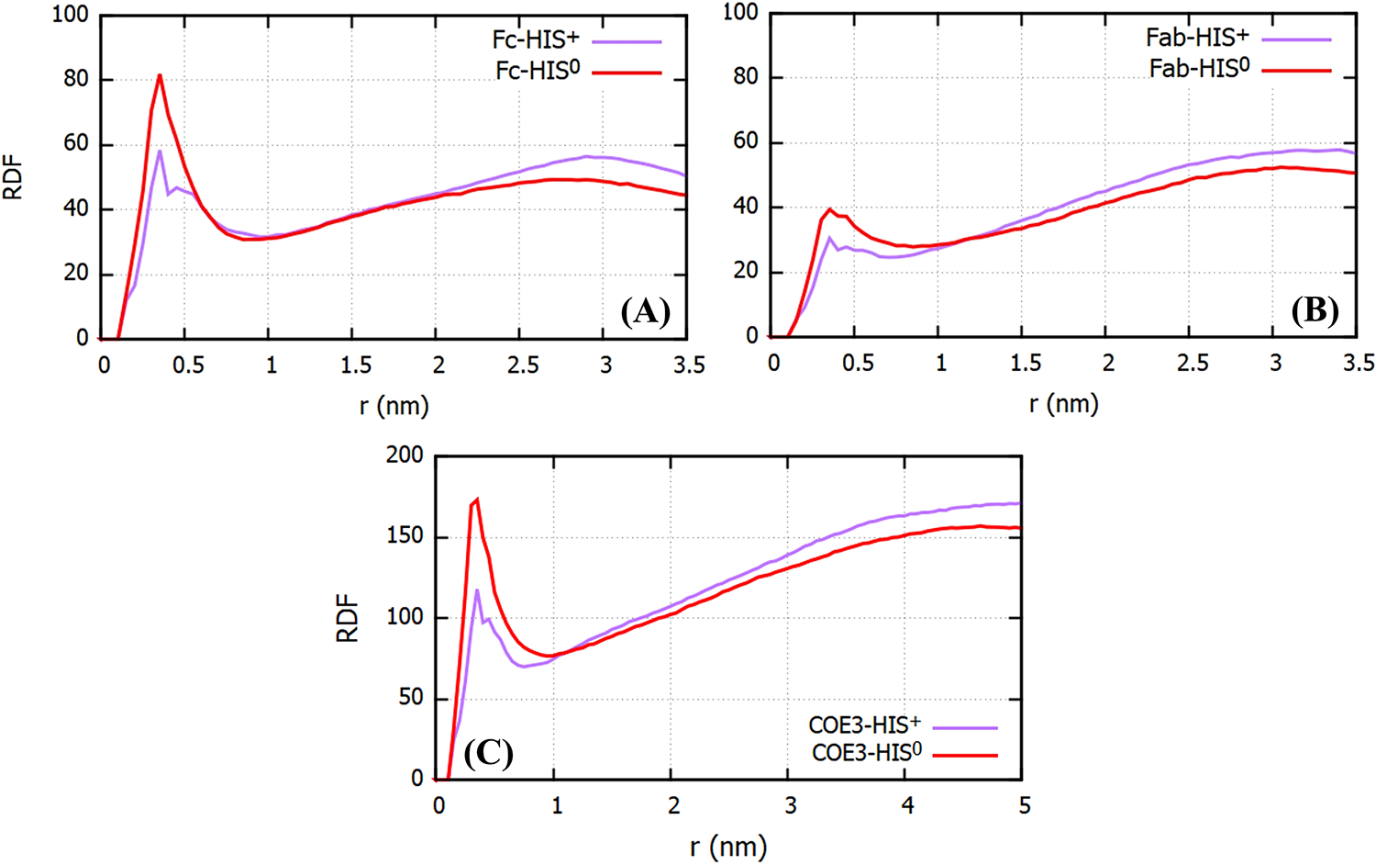
RDF for HIS^0^ and HIS^+^ as a function of distance measured from the surface of (A) Fc and (B) Fab fragments and (C) COE3. The value for each distance is obtained by averaging over three independent simulations (four in the case of COE3). The distributions were calculated using the gromacs tool *gmx rdf*, which calculates the number of atomic pairs in bins around the protein surface and divides the number of pairs by the bin width.

To assess the impact of histidine adsorption on the effective size of the protein, we computed *R*_g_ including the histidine molecules within the first solvation shell of the fragments and the antibody. The solvation shell can be identified by computing the radial distribution function (RDF) with respect to the surface of the antibody (see Figure 7). We identify the distance for the histidine-protein solvation shell with the first minimum in the RDF, ∼1 nm. Previous experiments reported a sharp increase in the hydrodynamic radius of the antibodies with increasing histidine concentration.^12^ However, we do not observe a noticeable increase in the protein size when we include the adsorbed histidines in our analysis (see Figure S3 of Supporting Information). We note that the hydrodynamic radius is an effective measure of the protein size and includes contributions from the surrounding solvent too. These contributions have not been considered in the calculations performed here. It would require more detailed analyses that take into account the correlations in the motion of the mAb and the surrounding water due to the presence of histidine.

### Histidine Adsorption on Fab and Fc Fragments

To investigate the buffer-protein contacts and the differences in the behavior of charged and uncharged histidines around the Fab/Fc fragments, we calculated the RDF of the HIS residues around the Fab/Fc fragments as a function of the distance from the protein surface (see Figure 7). Histidines show stronger adsorption on the Fc surface as compared to Fab. Also, the HIS^0^ residues show stronger adsorption than HIS^+^ on both the Fab and Fc fragments. The stronger adsorption of HIS^+^ on the Fc fragment is consistent with the fact that the Fc fragment has a smaller net + ve charge as compared to Fab; hence, HIS^+^ experiences a stronger electrostatic repulsion from the Fab fragment. The stronger adsorption of HIS^0^ as compared to HIS^+^ can be rationalized using a similar electrostatic argument. Because of its charge, HIS^+^ experiences strong repulsion from the ARG and LYS residues while the interaction of HIS^0^ with ARG and LYS is attractive, and the expected stabilization energy is ca. −5 kcal/mol.^41^

**Figure 8:**
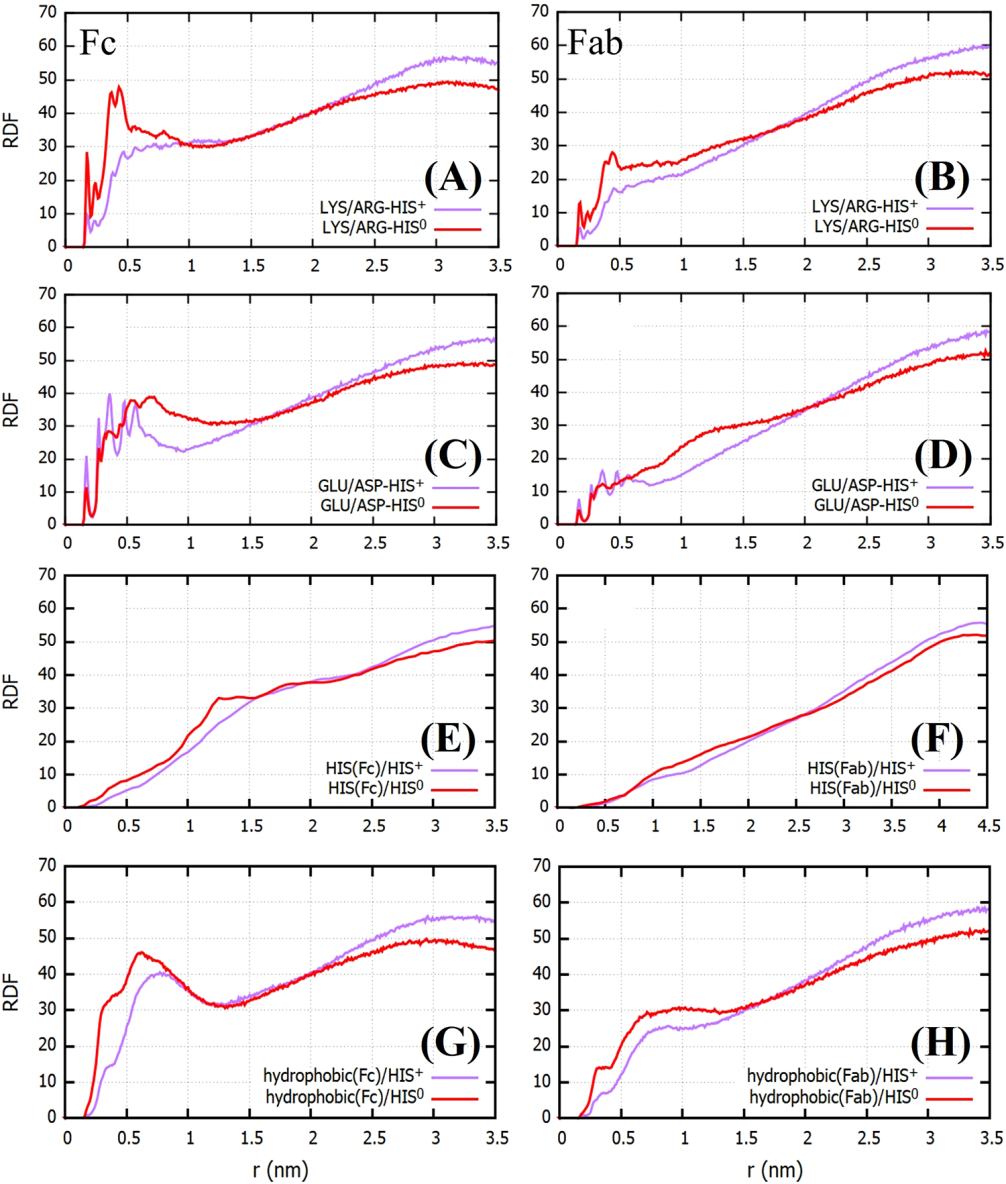
RDFs of HIS^0^ and HIS^+^ as a function of distance measured from regions of the Fc and Fab surface containing (A, B) positively charged residues (LYS/ARG), (C, D) negatively charged residues (GLU/ASP), (E, F) histidine residues, and (G, H) hydrophobic residues. The RDFs shown were obtained by averaging over three independent trajectories.

We show in Figure 8A–D the RDFs of histidines around the charged residues (ARG, LYS, GLU, and ASP) of the Fab and Fc fragments. Owing to the attractive interaction between HIS^0^ and the positively charged amino acids, we observe a more prominent peak in the LYS/ARG-HIS^0^ distribution as compared to HIS^+^. While we observe slightly higher low distance peaks for GLU/ASP-HIS^+^ pairs (see Figure 8C,D), the distribution of buffer molecules around these negatively charged residues indicates weak adsorption for both HIS^0^ and HIS^+^, which is evident when comparing the RDF around GLU/ASP with LYS/ARG. Histidine is known to interact favorably with other histidine residues in a protein.^41^ The HIS(Fab/Fc)-HIS^0/+^ RDFs, however, show a negligible interaction of the buffer histidines with the histidines on the protein surface (see Figure 8E,F). In contrast, the RDF of buffer histidines around the hydrophobic residues (Leu, Ile, Pro, Cys, Val, Tyr, Trp, Met, and Ala) indicates considerable affinity of the histidines for the hydrophobic regions of the proteins, especially in case of the Fc fragment. The main peak of the RDF corresponding to the hydrophobic regions is higher than the peak around the negatively charged regions. As discussed later, the apparent affinity of the buffer histidines toward the hydrophobic regions has implications for the aggregation properties of the proteins.

To understand the impact of ionic strength on the histidine adsorption on the proteins, we calculated the RDFs between histidine and NaCl (see Figure S4 in the Supporting Information). The RDFs show significant prominent peaks at short distances, indicating strong interactions between COO^–^ and NH_3_^+^ groups with Na^+^ and Cl^–^, respectively. However, the coordination number associated with the main peaks C(COO^–^)-Na^+^ and N(NH_3_^+^)-Cl^–^ is low (<0.1), showing that most histidines are not interacting directly with NaCl. Hence, we expect minor differences in the adsorption behavior of HIS in the presence or absence of NaCl. To test this idea, we performed additional simulations of Fab and Fc at free salt conditions (no NaCl, but counterions were present to ensure electroneutrality) and calculated the Fc-HIS and Fab-HIS radial distribution functions. The results shown in Figure S5 of the Supporting Information show that the histidine distribution around the protein does not depend significantly on the presence of salt. Hence we conclude that adding 0.15 M NaCl in the formulation does not modify histidine adsorption substantially.

We have used electrostatic arguments to explain the stronger adsorption of the histidine molecules on the Fc surface and the stronger adsorption of HIS^0^ on the Fab/Fc surface as compared to HIS^+^. However, it is not obvious why neutral HIS^0^ adsorbs more strongly on Fc than Fab (cf. top panel of Figure 7 and Figure 8A,B). To rationalize this behavior, we need to evaluate the relative contribution from nonelectrostatic interactions toward the adsorption of HIS^0^ on the Fab/Fc surface. We address this point in the next section.

### Quantitative Analysis of Histidine Fab/Fc Interactions

**Figure 9:**
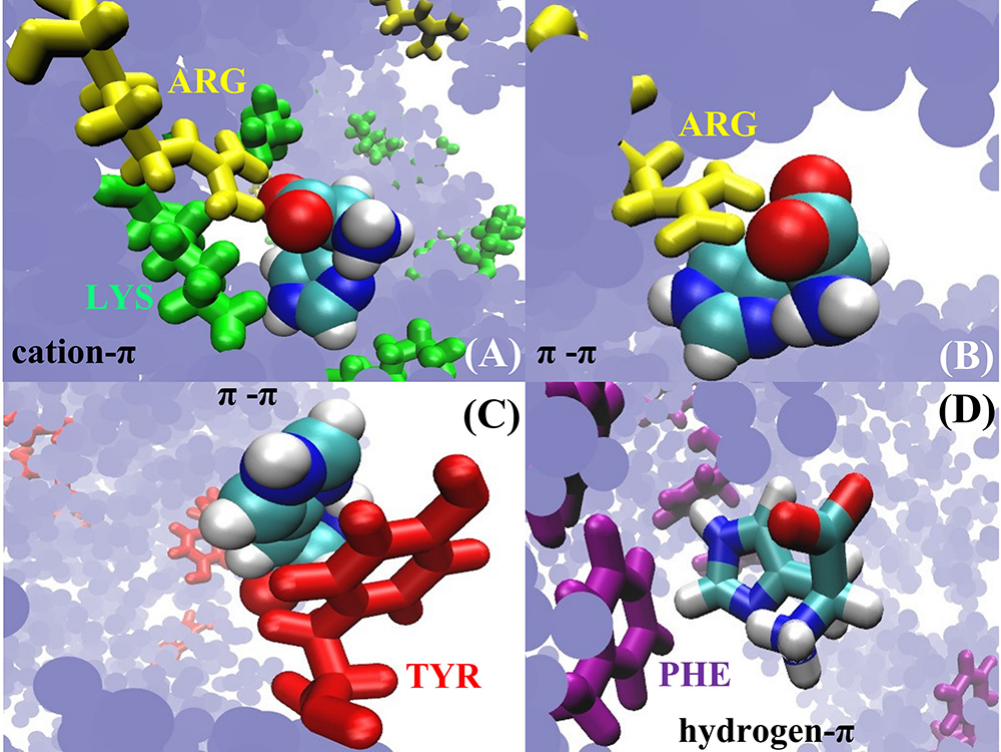
Histidine interaction types: (A) cation−π interaction between LYS (green) and HIS^0^. HIS^0^ interacts with a nearby ARG (yellow) residue through its negative terminus. (B) Illustration of concurrent electrostatic and π–π interaction between ARG and HIS^0^. (C) π–π interaction between HIS^0^ an a TYR residue (red). (D) An HIS^0^ residue interacting with the π cloud of a PHE residue (purple) with the plane of the histidine ring perpendicular to the PHE ring. The hydrogen-bond donating group in histidine points toward the center of the PHE ring. The blue background represents the other amino acids in the protein.

Histidines engage in four main kinds of interaction with other amino acid residues in a protein:^41^ (i) cation−π, (ii) π–π stacking, (iii) hydrogen−π, and (iv) hydrogen bonding. We expect the same set of interactions to be present between the amino acids on the protein surface and the surrounding buffer histidines. Here, we compare the interaction of HIS^0^ with Fab and Fc fragments with respect to the above-mentioned interaction types. For HIS^0^, the interaction type (i) involves positively charged residues (ARG, LYS, and HIS^+^ of the Fab/Fc domain) as interaction sites. Interaction type (ii) requires either aromatic residues, ARG, or histidines belonging to the protein interacting with HIS^0^. For type (iii), HIS^0^ interacts with aromatic residues of the protein, and for interaction type (iv) HIS^0^ forms hydrogen bonds with the hydrophilic amino acids on the protein surface, with its polar NH group acting as a donor and the electronegative N atom of the ring acting as an acceptor. Instances of these interactions from the MD trajectory are shown in Figure 9.

We introduce in the following a parameter, *S*_E_, to quantify the affinity of the Fab/Fc surface toward HIS^0^. The parameter is defined as
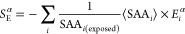
6where *E*_*i*_^α^ is the interaction energy between HIS^0^ and the amino acid type *i* (belonging to Fab or Fc) for interaction type α. The negative sign in eq (6) results in an index with higher positive scores for stronger attractive interactions. ⟨SAA_*i*_⟩ represents the combined solvent-accessible surface area of all the atoms belonging to amino acid type *i* averaged over the MD trajectory. SAA_*i*(exposed)_ is the solvent-accessible surface area of the side-chain atoms of amino acid type *i* in a solvent-exposed state. To calculate SAA_exposed_ of the side chain of an amino acid type *i*, we use the approach introduced in the work by Chennamsetty et al.^17^ The amino acid is considered part of the Ala–*i*–Ala trimer, and the SAA of the amino acid *i* is calculated in water. Throughout the simulations, the C atom of the carboxyl terminus and the N atom of the amide terminus were held fixed to their starting positions using harmonic restraints of force constant 1000 kJ/(mol nm^2^) to simulate the fully extended conformation of the trimer. We performed 50 ns long simulations of the trimer with *i* corresponding to each of the 20 amino acids and calculated the average SAA of the side-chain atoms of *i* over the trajectory to obtain SAA_*i*(exposed)_ (see Table S1 of Supporting Information for the SAA_*i*(exposed)_ values). For a given interaction type, a higher positive value of *S*_E_ for a protein indicates that the protein contains a greater number of solvent-exposed interaction partners of HIS^0^, which would then lead to a stronger interaction between HIS^0^ and the protein surface.

To quantify the hydrogen-bonding interactions between the proteins and HIS^0^ we introduce a second scoring function
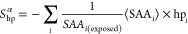
7where hp is the Black and Mold (BM)^42^ hydrophobicity for residue type *i*. The BM scale is shifted such that GLY has a hydrophobicity equal to 0. All the SAA and hydrophobicity parameters used in the calculations presented in this work are listed in Table S1 of the Supporting Information.The partial scores contributed by a particular interaction pair (residue-HIS^0^) and a particular interaction type, and the cumulative scores for each interaction type for the Fab and Fc domains, are listed in Table 2. The score, *S*^α^, for the Fc fragment is greater than that of the Fab for each type of interaction. For the cation−π interaction, Lys is the dominant contributor for Fc and Fab. While the energy of interaction of these amino acids with HIS^0^ is comparable, LYS is more exposed to the solvent, leading to a larger score. ARG is hydrophilic in nature, but the π cloud of its guanidinium group interacts strongly with the histidine ring leading to a high π–π interaction score. For h−π interactions, the Tyr–HIS pair clearly dominates for both Fc and Fab. For the h-bonding interaction, LYS is again the dominant contributor owing to its larger exposure to the solvent. Comparing the Fab and Fc fragments, the differences in scores arise from the cation−π, h−π, and the h-bonding interaction with an almost equal π–π interaction score. The higher value of *S*^α^ for Fc for all the different α values shows that, as compared to Fab, the Fc surface features a larger number of favorable HIS^0^ interaction sites, hence providing a quantitative explanation for the larger affinity of HIS^0^ for Fc (see Figures 7 and 8 and compare with the same results for Fab).

**Table 2:** HIS^0^ Interaction Scores for Fab (*S*_Fab_^α^) and Fc (*S*_Fc_^α^) Fragments^a^

interaction type (α)	*E*_int_^α^	HP	(SAA)_Fc,avg_^*i*^	(SAA)_Fab,avg_^*i*^	(SAA)_exposed_^*i*^	(*S*_Fc_^α^)^*i*^	(*S*_Fab_^α^)^*i*^
**cation–**π**:**							
Arg	–8.193		12.9	12.5	2.22	47.7	46.1
Lys	–9.268		34.5	28.3	1.93	165.3	135.5
total (S^α^)						213	181.6
π–π **stacking:**							
Phe	–0.093		4.7	2.5	1.9	0.2	0.1
Tyr	–0.098		9.4	9.5	2.0	0.5	0.5
Trp	–0.535		0.7	0.3	2.3	0.2	0.1
Arg	–2.402		12.9	12.3	2.2	14.0	13.3
total (S^α^)						15	14
**h–**π**:**							
Phe	–2.735		4.7	2.5	1.9	6.8	3.6
Tyr	–2.599		9.4	9.5	2.0	12.3	12.4
Trp	–3.679		0.7	0.3	2.3	1.1	0.5
total (S^α^)						20.2	16.5
**h-bonding:**							
Arg		–0.50	12.9	12.5	2.2	2.9	2.8
Asn		–0.27	17.7	8.4	1.4	3.5	1.7
Asp		–0.47	9.9	8.2	1.2	3.9	3.3
Gln		–0.25	17.6	11.2	1.6	2.7	1.7
Glu		–0.46	20.2	9.7	1.5	6.1	2.9
Lys		–0.22	34.5	28.3	1.9	3.9	3.2
Ser		–0.14	13.9	34.9	1.0	2.0	5
Thr		–0.05	9.7	19.3	1.2	0.4	0.8
His		–0.34	9.7	2.1	1.6	2	0.4
total (S^α^)						27.4	21.8

a The interaction energies, *E*_int_^α^ (in kcal/mol), are taken from ref (41). α is the interaction type, and *i* refers to the amino acid type. HP is the residue hydrophobicity taken from the Black and Mold scale.^42^ (SAA)_Fab,avg_ and (SAA)_Fc,avg_ are the average solvent-accessible surface areas of different residue types in the Fab and Fc domain, respectively. (SAA)_exposed_ is the solvent-accessible area for different residue types (*i*), calculated using the Ala–*i*–Ala tripeptide in pure water.

Note that there are several interactions that we have not included in our calculation. For instance, the buffer HIS^0^ and the histidines in the protein can also exhibit π–π stacking. In addition, two histidine molecules can coordinate, through their basic imidazole nitrogen, with the same metallic cation and form an ion-mediated interaction pair. Such ion-mediated interactions may exist between the buffer histidines and the histidines on the protein surface. Including these interactions would not change the result, given that the number of solvent-exposed HIS residues in the Fc fragment is larger than those in Fab (indicated by the values of the SAA for HIS in Table 2). This conclusion is supported by the negligible adsorption of the buffer histidines around the histidines in the Fab/Fc fragments (see Figure 8E,F).

### Free Energy of Histidine Binding

**Figure 10:**
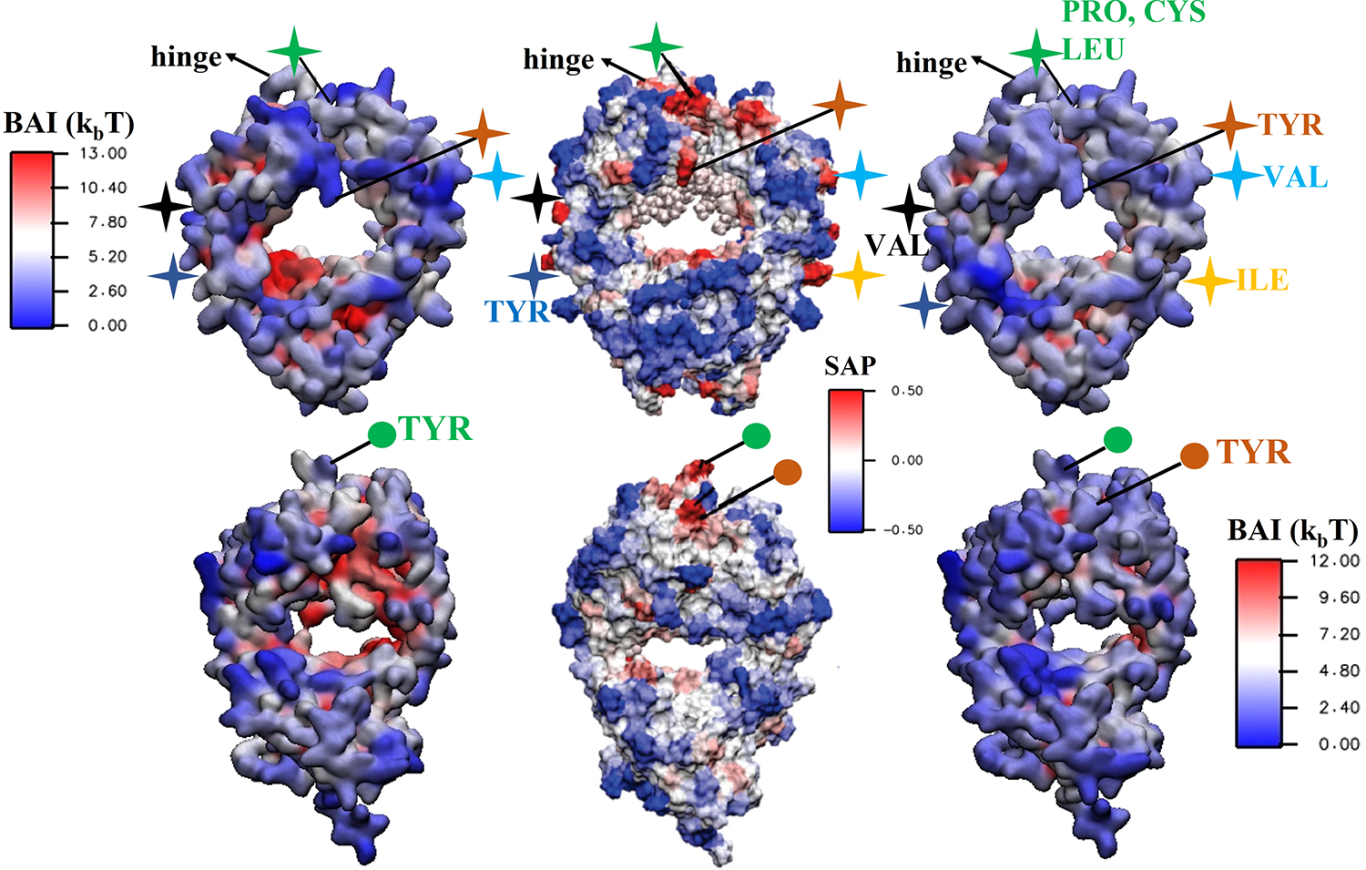
BAI obtained with eq 8 and represented as a color plot on the surface of Fc (top) and Fab (bottom) fragments, for HIS^+^ (left) and HIS^0^ (right) buffer molecules. A lower BAI value corresponds to a higher number of contacts between the protein and the buffer. We compare the BAI index with the SAP color plot (middle panel) for the same proteins (Reprinted adapted with permission from ref (44). Copyright 2010 American Chemical Society). Equivalent regions on the BAI and SAP plots are indicated by stars (for Fc) and circles (for Fab) of the same color. HIS interacts strongly with aggregation-prone regions, shown in red, on the SAP plot (middle panels) and blue in the BAI color plots (left and right panels).

**Figure 11:**
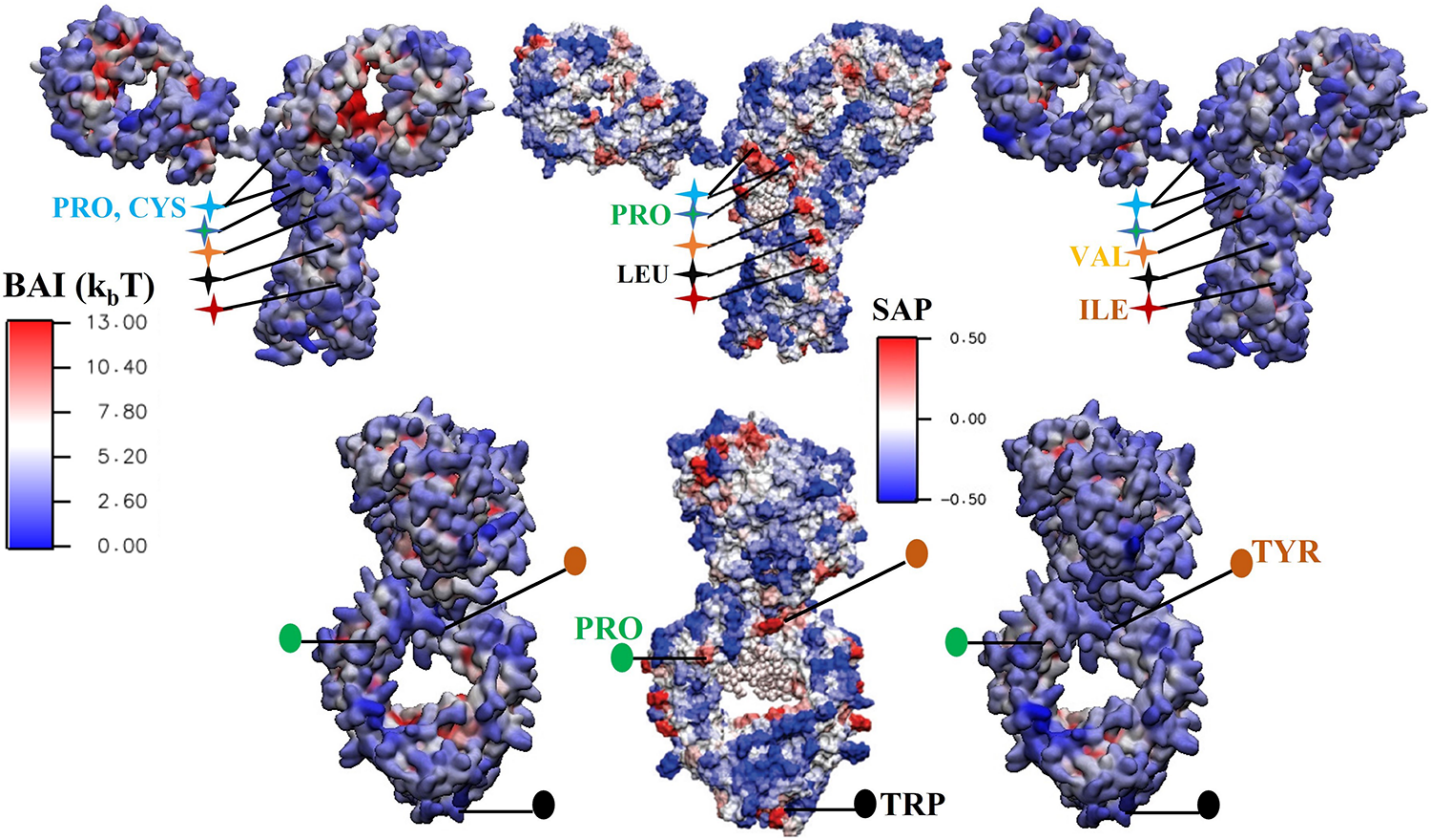
BAI obtained with eq 8 and represented as a color plot projected on the surface residues of the COE3 antibody. We show results for HIS^+^ (left) and HIS^0^ (right) buffer molecules, and these are compared with the SAP color plots (middle panel) for the same protein (Reprinted adapted with permission from ref (44). Copyright 2010 American Chemical Society). The top and bottom panels correspond to the front and side views. Equivalent regions on the BAI and SAP plots are indicated by stars (for the front view) and circles (for the side view) using the same color. The aggregation-prone regions (shown in red) on the SAP plot feature the strongest adsorption of the histidine buffer (see BAI plots).

To identify the most prominent histidine-binding regions on the surface of Fab and Fc fragments as well as the relative importance of different surface residues with respect to histidine binding, we computed the relative residue-level free energy of binding on the protein surface using the following procedure. For each of the three independent trajectories of Fab, Fc, and COE3, we calculated the number of atomic contacts (*N*^*i*^) between each of the protein residues (*i*) and the buffer histidine molecules. We defined a contact to exist between a protein residue and a buffer histidine molecule if the minimum distance (*d*_min_) between any atom of the residue and any atom of the buffer histidine is at most 0.4 nm (see Materials and Methods). The number of atomic contacts (*N*^*i*^) between residue *i* and the buffer (including all buffer molecules) is then equal to the number of intermolecular (buffer-residue) atomic pairs with distance less than or equal to 0.4 nm. The calculations were averaged over the three independent trajectories to obtain *N*_avg_^*i*^. We define the Buffer Adsorption Index (BAI) as
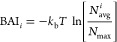
8which quantifies the relative free energy of forming contacts with buffer histidines, for each amino acid residue of Fab, Fc, or COE3. Here *N*_max_ is the largest value among all *N*_avg_^*i*^ values and corresponds to the protein residue showing the highest affinity for histidine adsorption. We performed the calculation separately for HIS^0^ and HIS^+^. *N*_max_ is the overall highest number of contacts irrespective of the buffer histidine charge state. This is done to ensure that we use the same energy reference for both HIS^0^ and HIS^+^. The residue types corresponding to *N*_max_ are ARG (for the Fc fragment) and LYS (for the Fab fragment and COE3). Both these residues are positively charged and strongly hydrophilic. Results for Fab, Fc fragments and COE3 are shown in Figure 10 and Figure 11 as color maps projected on the surface of the proteins. These maps reveal large variability in the free energy landscape of buffer adsorption, with significant differences in free energy, ∼12–13*k*_B_*T* units for Fc and Fab, and ∼16*k*_B_*T* units for COE3, between residues featuring the largest and smallest number of contacts. The heterogeneous free energy landscape is consistent with previous simulations of histidine-antibody interactions, which revealed different diffusive behaviors of the HIS molecules, representative of strong, weak, and no-adsorption states.^43^ Furthermore, this work demonstrated the stereospecificity of the interactions between histidine and the antibody.

The spatial aggregation propensity^17^ parameter is often used to identify aggregation-prone regions on the surface of proteins. This atomic-level parameter provides information on the amino acids that can be mutated to increase the stability of therapeutic proteins against aggregation. The SAP for an atom *j* can be calculated using the equation
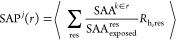
9where the sum runs over all the residues (res) with at least one side-chain atom within a radius *r*, taken here as 0.5 nm, from atom *j*. The brackets indicate an ensemble (time) average. SAA^*k*∈*r*^ is the combined solvent accessible area of all side-chain atoms *k*, belonging to res, that lie within a distance *r* from *j*, and SAA_exposed_^res^ is the combined solvent-accessible surface area of all the side-chain atoms in the residue res fully exposed to the solvent. A fully exposed residue is defined as a residue res within the Ala–res–Ala trimer. *R*_h,res_ is the residue hydrophobicity following the Black and Mold scale.^42^ The BM scale is shifted such that *R*_h,GLY_ = 0 (see Table S1 of Supporting Information). For a given atom, *j*, the SAP^*j*^ is a sum of the total hydrophobicity in the region surrounding the atom, weighted by an SAA-dependent factor that quantifies the exposure of that region to the solvent, hence providing a measure of the solvent-exposed hydrophobicity around an atom. A +*ve* (−*ve*) value of SAP implies a net hydrophobic (hydrophilic) environment on the protein surface in the region around an atom. The residue SAP is the average of all the constituent atoms’ SAPs. We compare the surface plot of BAI with the surface plot of SAP in Figures 10 and 11 to gauge the aggregation propensity of the regions to which histidine binds. We find that the largest changes to the SAP occur for the residues that have a net hydrophilic amino acid environment around them (SAP < 0). However, histidine also shows significant adsorption in many regions that the SAP analysis identifies as hydrophobic and solvent-exposed (SAP > 0) and therefore prone to aggregation (see labeled regions in Figures 10 and 11. One such region is the part of the Fc domain corresponding to the COE3 hinge region (see Figure 10). The SAP color plot shows an aggregation-prone domain in red in the hinge region, and the BAI plot shows that buffer histidine, especially HIS^0^, interacts strongly with this region. A similar conclusion can be drawn by comparing the BAI and SAP for COE3 (see Figure 11). On the basis of SAP, the region near the hinge (labeled by blue-green +) is strongly aggregation-prone. The BAI color plots indicate that the region has a strong affinity toward buffer histidines, especially HIS^0^. In addition to the hinge, there are other regions on the surface of these proteins that are aggregation prone and also show affinity toward the buffer molecules. For instance, the antigen-binding region on the Fab fragment (Figure 10, labeled by orange ●) is centered around a TYR residue and is aggregation-prone. Similar to the hinge region, we observe significant histidine adsorption in this region. Hence we conclude that the buffer molecules bind favorably onto solvent-exposed hydrophobic regions on the mAb surface, such as the hinge region. The interaction of histidines with the hydrophobic regions of the protein can be through intermittent direct interactions with the hydrophobic amino acids or through persistent electrostatic interactions with neighboring charged amino acids, leading to shielding of the hydrophobic regions owing to histidine’s spatial extension.

Our analysis provides insight into the mechanism by which histidine might reduce mAb aggregation, as reported in experiments.^9^ Protein aggregation proceeds through various pathways. The Lumry-Eyring framework^45−47^ depicts the aggregation process initiating from a transition of the native protein structure into a partially unfolded aggregation intermediate, passing through a transition state. This reversible unfolded intermediate contains solvent-exposed hydrophobic patches and would form irreversible dimers with other unfolded mAbs. These dimers would then act as the nucleus for larger aggregates. In contrast, there are instances when native proteins form reversible encounter complexes that undergo a transition to irreversible dimers through structural changes, following complexation.^48^ Thus, aggregation is driven by partial unfolding and exposure of sequestered hydrophobic residues to the solvent. Association of folded mAbs to form reversible complexes is one of the means through which the mAb may unfold. If histidine molecules shield solvent-exposed hydrophobic regions (which act as centers for mAb association) on natively folded mAbs, the possibility of the mAbs forming encounter complexes would be reduced. Similarly, the shielding of hydrophobic patches on the aggregation intermediates (partially unfolded mAbs) would arrest the growth of the aggregates. This is consistent with experimental observation.^9^ Apart from shielding the hydrophobic regions on the protein surface from water, histidines, being hydrophilic in nature (a BM index of −0.34 as compared to a value of −0.5 for ARG, which is the most hydrophilic amino acid on this scale), would reduce the net hydrophobicity of the region to which they adsorb. The binding propensity of histidine to different types of hydrophobic amino acids on the Fab and Fc surface is shown in Figure S6 of the Supporting Information.

The histidine binding pockets highlighted in ref (43) are concentrated on the Fab domain of the mAb. In contrast, we observe histidine binding on the Fc domain as well. These differences are expected to be linked to the amino acid distribution at the surface of the mAbs used in their study compared to ours. However, the amino acids involved in binding to histidine are similar to what we observe in our simulations. We have added snapshots of the histidine binding pockets on the Fc surface in Figure S7 of the Supporting Information. The pockets mostly consist of a combination of charged and hydrophobic amino acids. The charged amino acids engage the NH_3_^+^ and COO^–^ groups of the histidines, while the hydrophobic amino acids interact with the histidine ring. The presence of charged amino acid residues near the surface of exposed hydrophobic ones enhances their shielding by histidine by electrostatically trapping the histidine in the vicinity of the hydrophobic residues. This notion might provide a design strategy to develop nonaggregating mAbs in histidine buffer.

**Figure 12:**
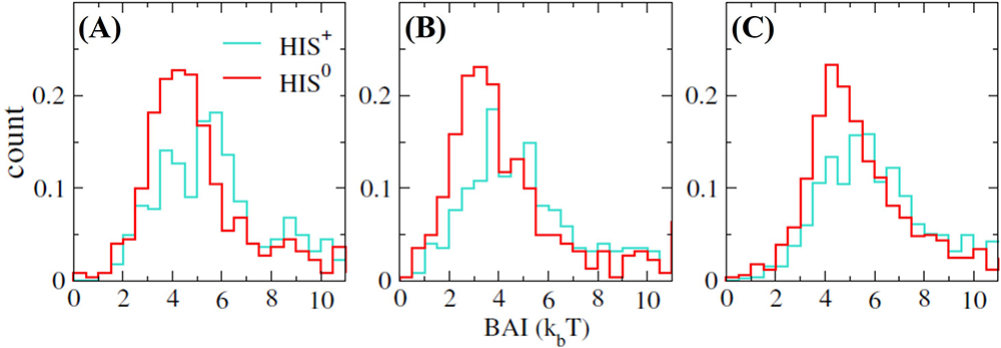
Normalized probability distribution of BAI for (A) Fc, (B) Fab, and (C) COE3. The distributions are represented in the range of BAI = 0–11 *k*_b_T. The full probability distribution contains contributions from protein residues that form no contacts with buffer histidine. See Figure S8 in the Supporting Information for a representation of the full distributions and the caption of that figure for additional details.

We focus now on the differences between the interaction of HIS^+^ and HIS^0^ with the proteins, by comparing the corresponding BAI indices. In Figure 12 we plot the distribution of BAI for the amino acid residues of Fc, Fab, and COE3. The maxima of the probability distribution for HIS^0^ are shifted toward a lower BAI index relative to HIS^+^. The positions of maxima for HIS^0^ and HIS^+^, for all three proteins, differ by ∼2*k*_B_*T*, which is significant as compared with the thermal energy. Furthermore, the average BAI given by the first moment of the distribution is lower for HIS^0^ (Fc: 6.8; Fab: 5.4; COE3:6.9) than HIS^+^ (Fc: 8.6; Fab: 7.0; COE3:8.6). These results indicate that there is a larger number of protein residues forming strong contacts with HIS^0^ as compared to HIS^+^. This result is consistent with the data shown in Figures 7 and 8 and supports the stronger affinity of HIS^0^ to adsorb on the protein surface observed in our simulations.

### A Protein Aggregation Index Including Buffer Effects

We now quantify the effect of histidine binding on the aggregation of the proteins. We use as a starting point the SAP index introduced in ref (17) (see eq 10) and extend the definition to incorporate the contribution from buffer histidines that adsorbed on the protein surface. We refer to this index as BSAP to highlight the fact that the buffer molecules adsorbed at the protein surface are included in the calculation. The BSAP index is defined as
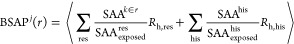
10The first summation is the same as in the SAP defined in eq (9). The main difference is that the SAA^*k*∈*r*^ is calculated by taking into account the presence of the buffer, whereby the contribution to the overall SAP of an atom *j* from its neighboring atoms will be lower if a buffer histidine is shielding the atoms from the solvent, since this results in a reduction in their SAA. The second summation contains contributions from the buffer histidines to the overall hydrophilicity around atom *j*. SAA^his^ is the solvent-accessible area for the buffer histidine molecule that has any of its atoms at a distance at most 0.4 nm from atom *j*, while SAA_exposed_^his^ is the accessible area of a bare histidine molecule in solution calculated by averaging over configurations from a 15 ns long simulation of a single histidine molecule (listed in Table S1 of Supporting Information). *R*_h,his_ is the BM hydrophobicity of histidine. The constituent terms for BSAP can be separated and written as follows.
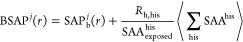
11Here the subscript, b, in the SAP term indicates that the surrounding buffer has been considered while calculating the SAA. As histidine is a hydrophilic amino acid, its presence near a hydrophobic residue would not only shield the residue from the solvent but also turn the region around the residue less hydrophobic. The details of the method used for the calculation are described in the Supporting Information (see Figure S9 and the accompanying text).

**Figure 13:**
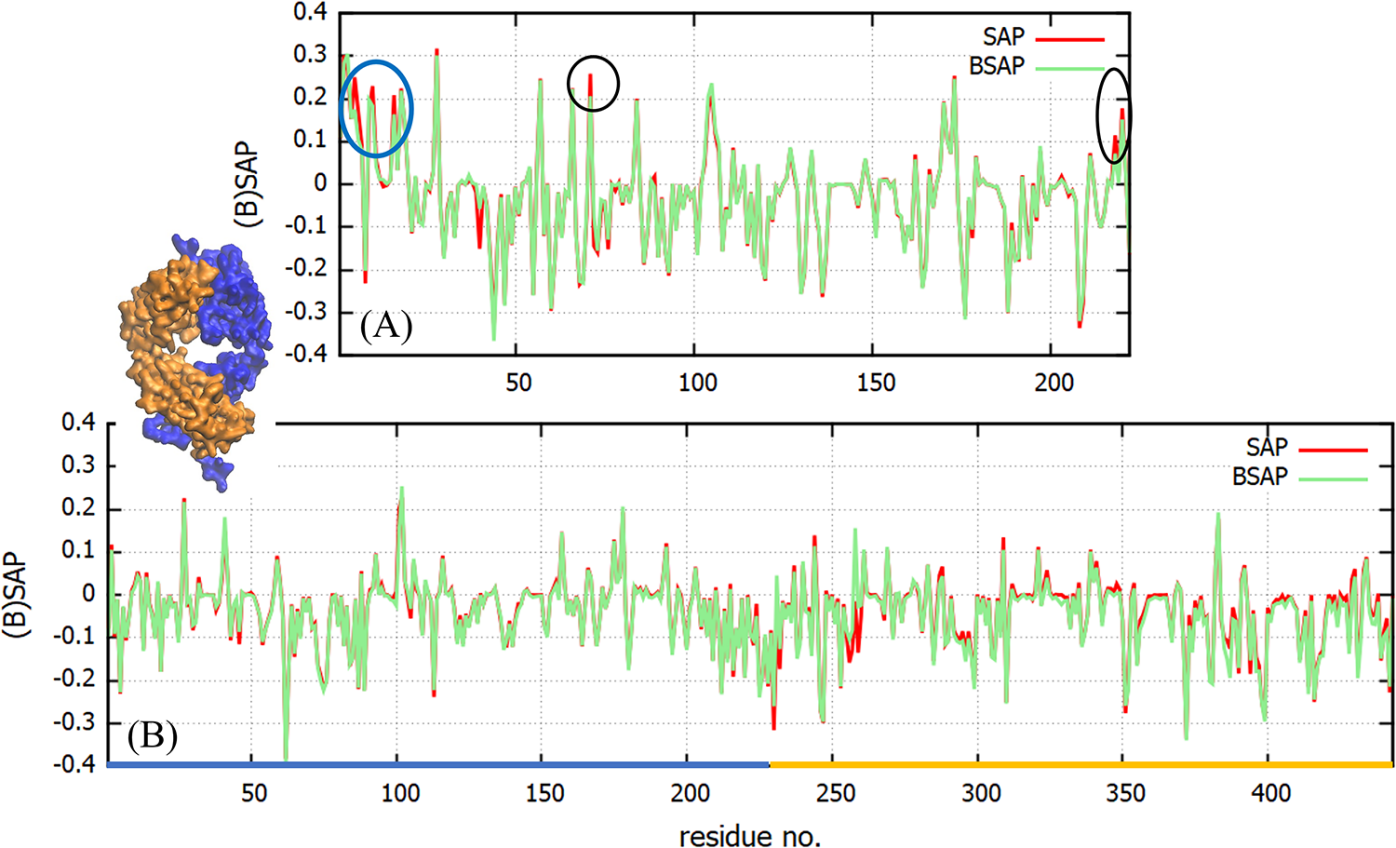
Comparison between the BSAP and SAP indices for the (A) Fc and (B) Fab fragments. The SAP parameter is obtained from trajectories at 0 mM HIS buffer. The blue and orange regions in the snapshot of the Fab fragment correspond to the heavy and light chain sections, respectively, and are also highlighted in the *x*-axes of the bottom panel. Areas with significant differences between the BSAP and SAP indices are identified with circles. The differences near the hinge region of the Fc are indicated by a blue circle.

The plots of the BSAP and SAP obtained for Fc and Fab are shown in Figure 13. We find that the largest differences between SAP and BSAP appear in the hydrophilic residues, which are larger in number and show greater affinity toward the histidine molecules. For the Fab fragment, we do not observe significant differences between the BSAP and SAP for the residues with SAP > 0 (see Figure 13). A plot for the difference between BSAP and SAP for each amino acid residue of the Fab and Fc fragments and the complete COE3 is shown in Figure S10 of the Supporting Information. For strongly hydrophobic regions near the hinge region of the Fc fragment (indicated with circles), we observe a reduction between 5 and 15% in SAP (see Figure 13A). We note that, while SAP is defined for each residue, it depends strongly on the nature of other amino acids, which are its close neighbors. Thus, a change in the value of SAP of a residue is a determinant not only of the affinity of histidine toward that residue but also the affinity toward neighboring residues, which might well include some aggregation-prone region of the protein surface. To assign an overall magnitude to the effect of histidines on the aggregation propensity for the proteins we evaluated the BSAP/SAP scores, by adding up all the positive atomic BSAP/SAP values, in the presence and absence of histidines
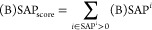
12where the sum runs over all the atoms, *i*, with SAP^*i*^ > 0. On the one hand, the SAP score for the Fc domain was 191.3 ± 4.6, while the BSAP score was found to be 182.5 ± 3.2. For the Fab fragment on the other hand the SAP score was 85.9 ± 0.08, while the BSAP score was found to be 74.1 ± 3.2. A calculation for the complete COE3 yields a similar trend with SAP and BSAP scores of 376.4 ± 7.4 and 354 ± 11.6, respectively. A reduction in BSAP score with respect to the SAP score amounts to a reduction in the overall exposed hydrophobicity of the proteins. This would reduce their tendency to associate and prevent the concomitant unfolding, leading to a reduction in protein aggregation. This result is consistent with the experimental observations.^9^

### Histidine Binding Kinetics

**Figure 14:**
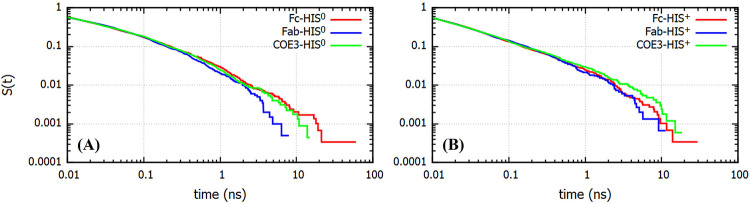
Time dependence of the survival probability (*S*(*t*)) for contacts between Fc/Fab/COE3 and (A) HIS^0^ and (B) HIS^+^.

To gain insight into the adsorption kinetics of histidine binding to the Fab/Fc fragments and the COE3 antibody, we calculated the survival probability *S*(*t*) (see Materials and Methods) for the binding process for both charge states of histidine. We show in Figure 14*S*(*t*) as a function of time for the binding of HIS^0^ and HIS^+^ with Fc, Fab, and COE3.

The *S*(*t*) for COE3-histidine binding was calculated from a single simulation performed using water molecules with their normal molecular mass (18 g/mol) unlike the structural analyses of COE3 reported in the previous section, which were performed using a rescaled mass for the water molecules (see Materials and Methods).

We quantify the rate of histidine detachment from the rate constant, *k*_off_, defined as the rate constant for the detachment of buffer histidine molecules adsorbed on the protein surface at time *t* = 0. The histidine molecule is deemed to be adsorbed on the protein surface if the closest protein-histidine distance is at most 0.4 nm (see Figure 4).

Previous studies have shown that the dynamics of water detachment from protein surfaces does not follow a simple exponential decay but a stretched exponential decay, indicating a non-Markovian process.^49^ To calculate the rate constants associated with the histidine desorption process from the protein surface we fitted the survival probabilities to a stretched exponential of the following form.
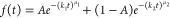
13The two terms are included to reproduce both the short and long time decays of the survival curves (see Figure 14). The exponents μ_1_ and μ_2_, listed in Table 3, measure the deviation of the desorption process from a pure exponential, μ = 1. The exponents were obtained from fits of the survival probabilities with eq 13. See figure S11 of the Supporting Information for a comparison of the fits with the simulated data.

**Table 3:** Histidine Desorption Rate Constants (*k*_1_ and *k*_2_) and Other Parameters Obtained by Fitting the Survival Probability with a Function of the Form Shown in eq 13^a^

interaction pair	*k*_1_ (ns^–1^)	*k*_2_ (ns^–1^)	μ_1_	μ_2_	⟨τ_r_⟩ (ns)
Fab-HIS^0^	49.28 ± 0.5	20.64 ± 0.25	0.62 ± 0.003	0.32 ± 0.001	0.13 ± 0.02
Fc-HIS^0^	45.03 ± 0.27	22.84 ± 0.18	0.66 ± 0.005	0.33 ± 0.001	0.18 ± 0.04
COE3-HIS^0^	45.9 ± 0.7	22.9 ± 0.5	0.6 ± 0.003	0.34 ± 0.002	0.14
Fab-HIS^+^	57.47 ± 0.47	26.06 ± 0.28	0.83 ± 0.008	0.34 ± 0.001	0.12 ± 0.01
Fc-HIS^+^	49.36 ± 0.5	34.32 ± 0.25	0.83 ± 0.003	0.31 ± 0.001	0.14 ± 0.04
COE3-HIS^+^	46.8 ± 0.16	39.4 ± 0.24	0.81 ± 0.002	0.28 ± 0.002	0.12

a The average residence times were calculated by averaging over temporal lengths of all the residence events occurring over the course of three independent MD runs. The error bars on the residence times were calculated over the average values calculated for each run. The residence time for COE3 has no error bars, as it was calculated from a single simulation. The error bars on the off-rates correspond to the standard error of fitting.

The desorption rates for HIS^0^ are always smaller than those for HIS^+^ indicating that the neutral histidine forms stronger contacts with the protein. Smaller values of μ_1_ for HIS^0^ indicate a larger deviation from exponential relaxation again indicating a stronger effect of the protein surface on the HIS^0^ dynamics as compared to HIS^+^. The typical relaxation times are between 17 and 20 ps for *k*_1_ and 25–50 ps for *k*_2_, indicating substantially different time-scales. The shorter times are similar to values observed before for the relaxation of water molecules at distances ∼0.4 nm from charged and polar protein sites.^49^ Overall, our results are consistent with the stronger adsorption of HIS^0^ on the protein surface (see Figure 8).

Table 3 also contains the average residence times of the binding events for different binding pairs (see Figure 4 for a definition of τ_r_). All the average residence times for HIS^0^ and HIS^+^ are on the order of 100 ps. These short residence times imply that the adsorption process is highly dynamic (see Figures S12 and S13 of Supporting Information) and that the adsorbed histidines, after short times, either diffuse back to the solution or diffuse on the surface of the protein undergoing intermittent detachments. The values for the Fc domain are slightly larger (∼1.5 times) than those for Fab and COE3. Both the rate constants and the average residence times include a contribution from long time scale binding events. Hence, the accuracy of the values would depend on the quality of statistics of these rare events. The importance of the rare-event statistics needs to be stressed, as the largest modifications in the aggregation behavior of the mAbs is expected to be affected by temporally long histidine adsorption events.

The adsorption of histidine is fairly heterogeneous (see Figures 10 and 11), and we expect that those histidines adsorbing strongly at the protein surface will feature slower dynamics. We performed computations of the survival probability function (see Figure 14) for histidine (HIS^0^) molecule showing very strong adsorption on the Fab surface (specifically at a binding pocket formed by ARG, GLU, PRO, and TYR, see also Figure S7 and Figure S14). The survival probability function, *S*(*t*), shows a significantly slower decay than the average *S*(*t*) reported in Figure 14. We have also calculated the *S*(*t*) for a histidine molecule that binds the protein surface for short times, which shows a significantly faster decay than the average. These results demonstrate that adsorption modifies the histidine dynamics significantly.

## Conclusion

We have performed large-scale MD simulations of the monoclonal antibody (mAb) COE3 in histidine buffer solutions. Our MD simulations of the mAb and its Fab and Fc domains provide insights into the buffer-protein interaction. The interaction depends very sensitively on the charge state of the buffer and, therefore, on pH. Neutral histidine (HIS^0^) shows a stronger affinity for adsorption on the protein surface than the positively charged histidine (HIS^+^). The adsorption of HIS^+^ is less favorable due to electrostatic repulsion from the positively charged proteins. We find a significant prevalence of adsorption on regions rich in hydrophobic amino acids, particularly in the hinge region of the antibody. While histidine adsorption has a negligible impact on the effective size of the protein, as quantified by the radius of gyration, it influences significantly the mAb hinge flexibility, which becomes stiffer in the presence of histidine buffer. We introduce contact-based free energy calculation to quantify the relative adsorption energy of histidine on the protein surfaces. Residue-wise free energies indicate that histidine has a strong affinity toward surface-exposed hydrophobic regions of the proteins. On the basis of our calculations we propose that the reduction in mAb aggregates in the presence of histidine is driven by shielding of the hydrophobic regions on the surface of native or partially unfolded mAbs. The shielding is affected by either direct short time-scale interactions with the hydrophobic amino acids or through long-lasting electrostatic interactions with charged amino acids that lie in the neighborhood of the aggregation-prone regions. Our work, thus, provides mechanistic insights into the experimentally observed influence of buffer composition on the aggregation of mAbs. Henceforth, existing aggregation metrics, such as SAP, might require an extension to include specific buffer effects. We introduce the BSAP index by incorporating the effect of buffer adsorption and hydrophilicity into SAP. We show that the new index predicts lower aggregation propensity for some aggregation-prone regions on the protein surface. We anticipate that the results presented here would guide future modifications in the local structure of mAbs (mutations) and formulation of buffer solutions aimed at reducing protein aggregation propensity.

While we have focused here on a set of relevant experimental conditions employed in pharmaceutical formulations, it would be very interesting to extend these studies to address different pH conditions. This might require using other buffers, for example, phosphate, to converge with experimental conditions. Similarly, it would be interesting to investigate the impact of thermal stress on histidine adsorption and protein stability. These topics might be suitable for extensions of our work, possibly considering other monoclonal antibodies.

## Supporting Information Available

Additional results on protein structures, radii of gyration as a function of time, histidine binding pockets, amino acid hydrophobicity, procedure for SAP and BSAP calculations, Survival probabilities of histidine-protein contacts (PDF)

## Notes

FORTRAN and bash scripts used to calculate the Spatial Aggregation Propensity available at: https://github.com/bresmegroup/Buffer-SAP_BSAP, https://github.com/bresmegroup/SAP-Protein-in-Histidine-Buffer, https://github.com/bresmegroup/SAP_bare-protein
