# Supplementary material for: Understanding the Stabilizing Effect of Histidine on mAb Aggregation: A Molecular Dynamics Study
Source: Mol Pharm. 2022 Aug 10;19(9):3288–303. doi: 10.1021/acs.molpharmaceut.2c00453 (PMC9449975; doi:10.1021/acs.molpharmaceut.2c00453)
Supplement: Supplementary file 1 — mp2c00453_si_001.pdf [file mp2c00453_si_001.pdf]

Understanding the stabilizing effect of histidine on mAb aggregation: A molecular dynamics study

Supplementary Information

Suman Saurabh,[†] Cavan Kalonia,[‡] Zongyi Li,[¶] Peter Hollowell,[¶] Thomas Waigh,[¶,§] Peixun Li,[‖] John Webster,[‖] John Seddon,[†] Jian R. Lu,[¶] and Fernando Bresme[*,†]

†*Department of Chemistry, Molecular Sciences Research Hub Imperial College, W12 0BZ, London, United Kingdom*

‡*Dosage Form Design and Development, BioPharmaceutical Development, BioPharmaceuticals R&D, AstraZeneca, Gaithersburg, Maryland 20878, United States*

¶*Biological Physics Group, School of Physics and Astronomy, Faculty of Science and Engineering, Oxford Road, The University of Manchester, Manchester M13 9PL, UK*

§*Photon Science Institute, The University of Manchester, M13 9PL, UK*

‖*STFC ISIS Facility, Rutherford Appleton Laboratory, Didcot, OX11 0QX, UK*

E-mail: f.bresme@imperial.ac.uk

Phone: +44 207 594 5886

Initial structures

(A)

(B)

(C)

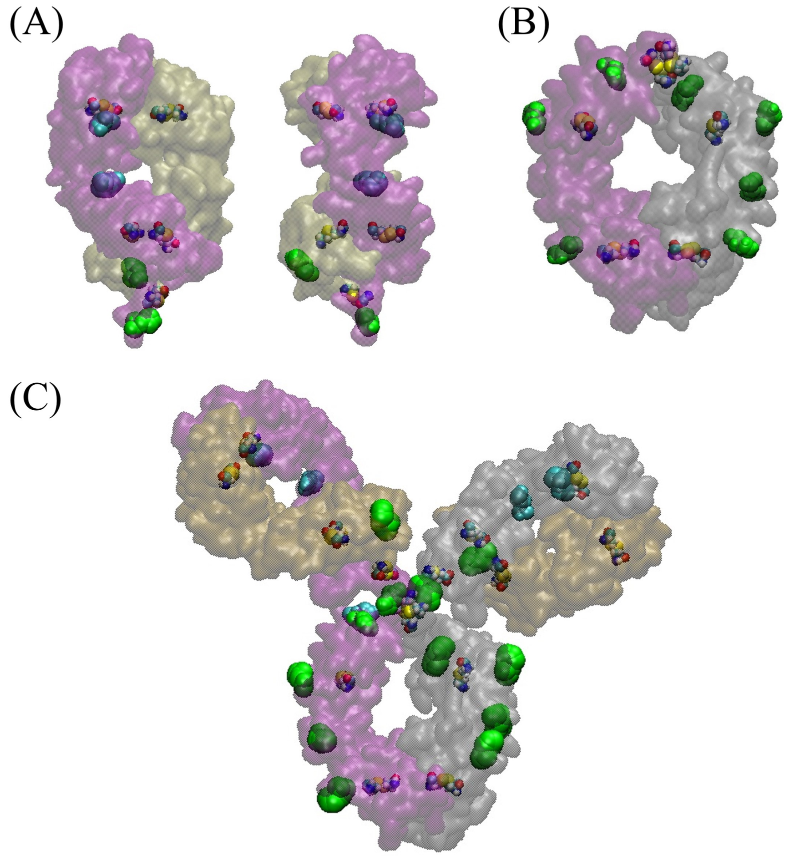

Figure S1: **The initial structures of the (A) Fab and (B) Fc fragments and the complete (C) COE3 antibody. The positively charged HIS and the neutral GLU residues of the proteins (as predicted by propKa3.0) are shown in green and blue respectively. The two light chains are depicted in tan and the two heavy chains are shown in purple and grey. The front and side views of the Fab domain are shown separately to help visualize the location of different charged histidines. The cystine residues forming the disulfide bonds are also shown (in yellow).**

Radius of Gyration of the Fab and Fc domains

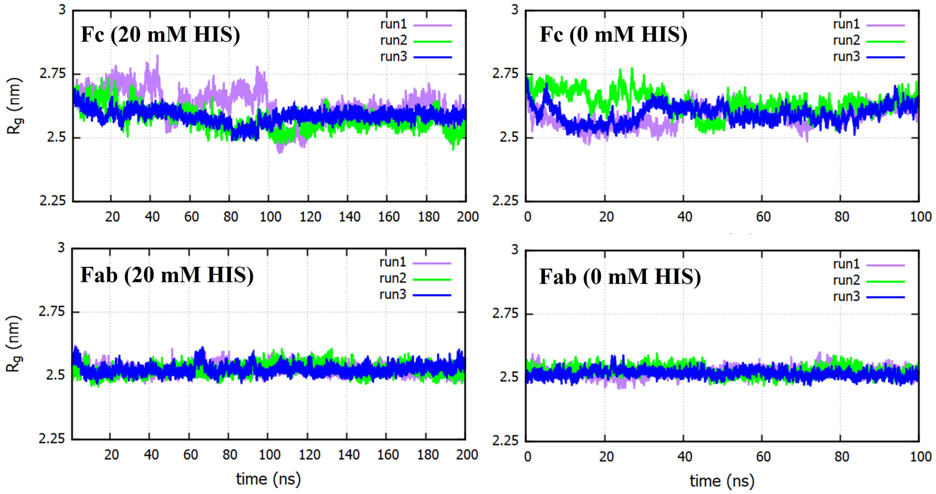

Figure S2: **Radius of gyration for the Fab and Fc domains for simulations performed in the presence and absence of histidine.**

Radius of Gyration of the Fab and Fc domains including coordinates of adsorbed buffer histidines

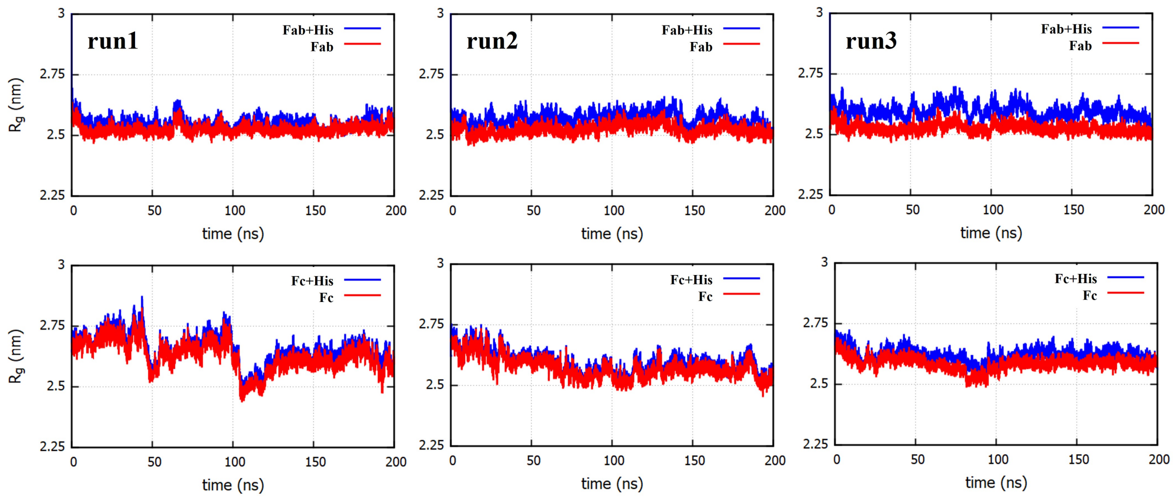

Figure S3: **Radius of gyration for the Fab and Fc domains including the coordinates of buffer histidines within the first adsorption shell (1 nm from the protein surface).**

Histidine-Salt interaction

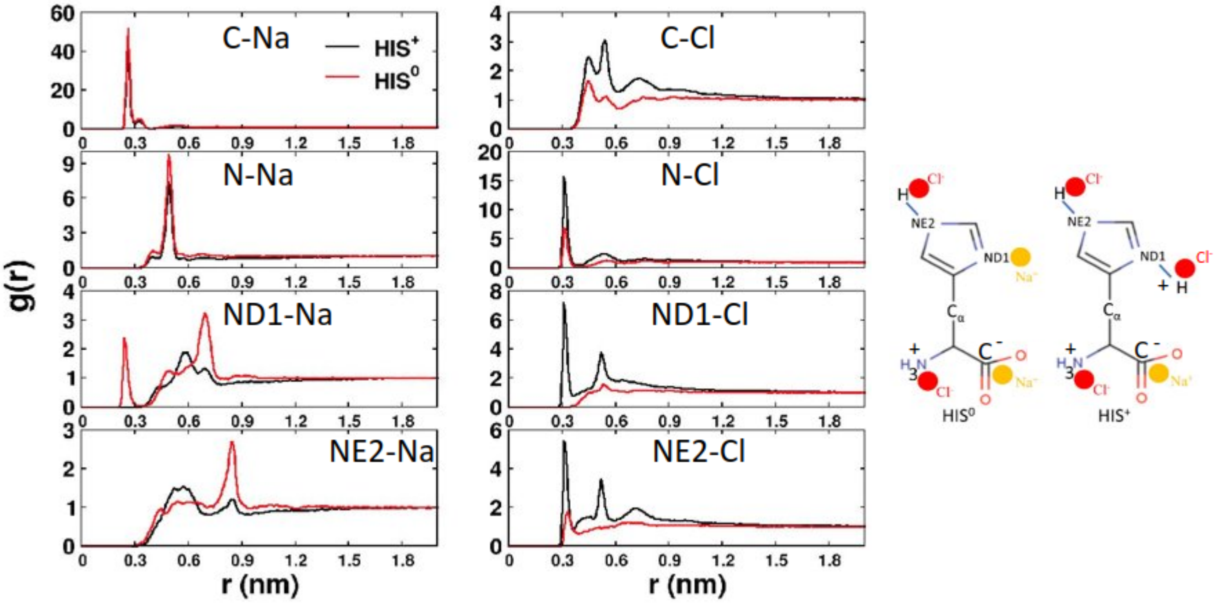

Figure S4: **Radial distribution of the Na^{+} and Cl^{-} around the N, NE2, ND1 and C atoms of HIS^{+} and HIS^{0}. The panel on the right indicates the pair interactions between the Na^{+} and Cl^{-} ions and the different sited in the histidine molecules.**

Histidine distribution around Fc and Fab: 150mM NaCl vs. 0 mM NaCl

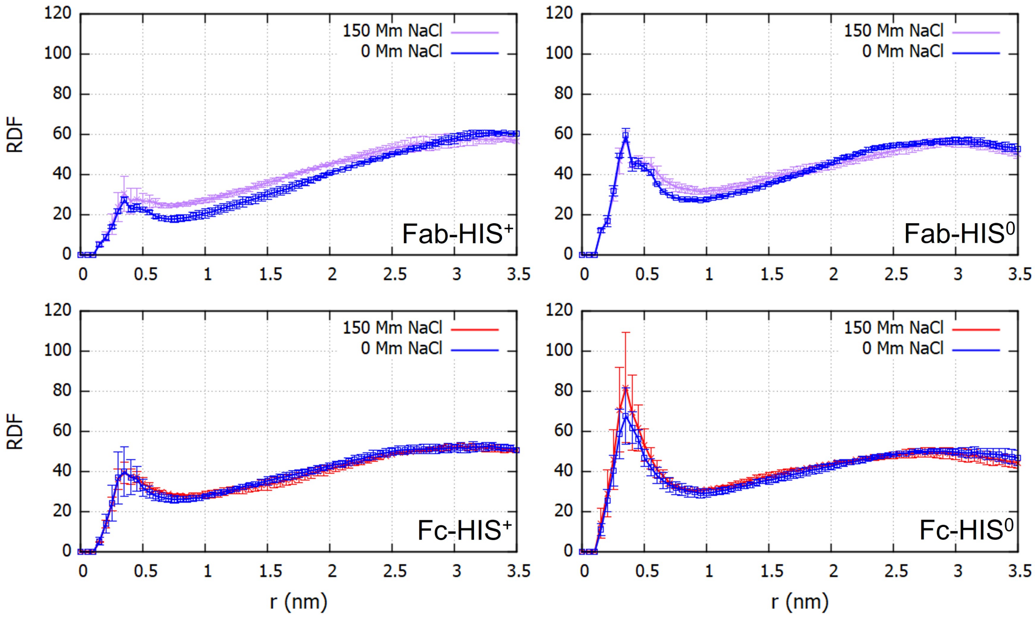

Figure S5: **The radial distribution of HIS^0 and HIS^+ in the absence of salt compared to the distribution at 150 mM NaCl.**

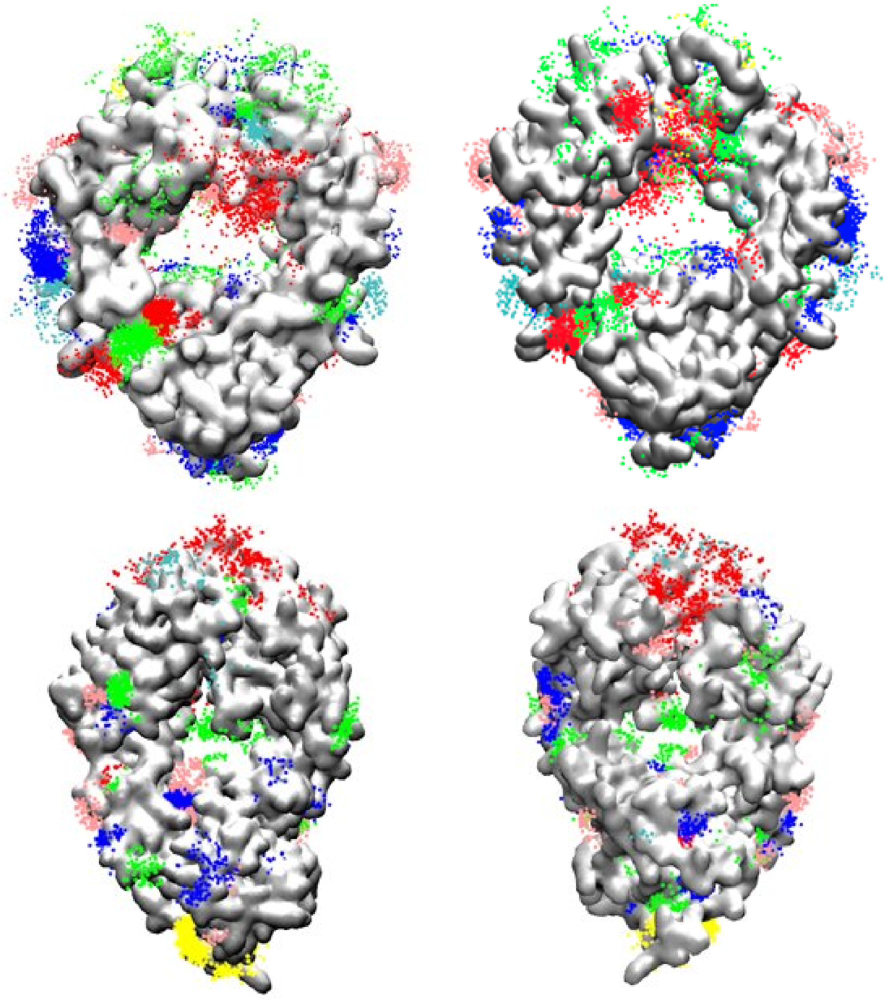

Figure S6: **The histidine cloud around the hydrophobic regions in Fc (top) and Fab (bottom) is depicted by visualizing for multiple frames the atoms belonging to the histidine residues within 0.3 nm of various hydrophobic amino acids. The clouds around TYR are shown in red, PRO in green, LEU in blue, ILE in cyan, VAL in pink and CYS in yellow. A high cloud density can be seen around some of these hydrophobic amino acids, and there is a larger cloud density on the Fc fragment compared to that on the Fab fragment. Among the hydrophobic amino acids, TYR, LEU, PRO and CYS form more contacts with histidine while TRP has the least number of contacts.**

Histidine-binding pockets

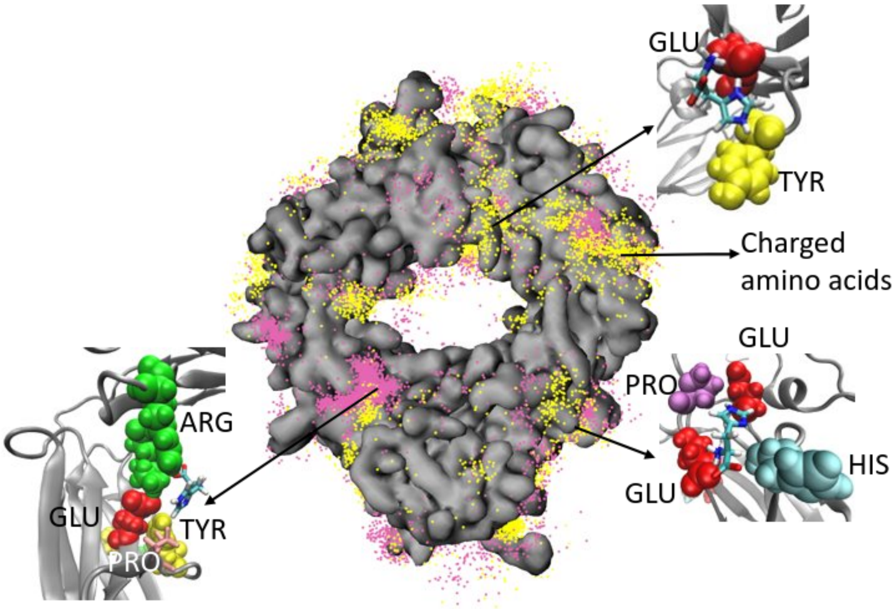

Figure S7: **Histidine binding pockets on the Fc domain, The mauve and yellow clouds correspond to the HIS^0 and HIS^+ atoms within 0.2 nm of the surface of the protein. A cutoff of 0.2 nm is used so that only the most prominent binding pockets are visualized. The atomistic structures of various binding pockets are shown. The binding pockets on the Fab domain are of a similar nature, consisting of a mixture of charged and hydrophobic amino acids.**

BAI distribution

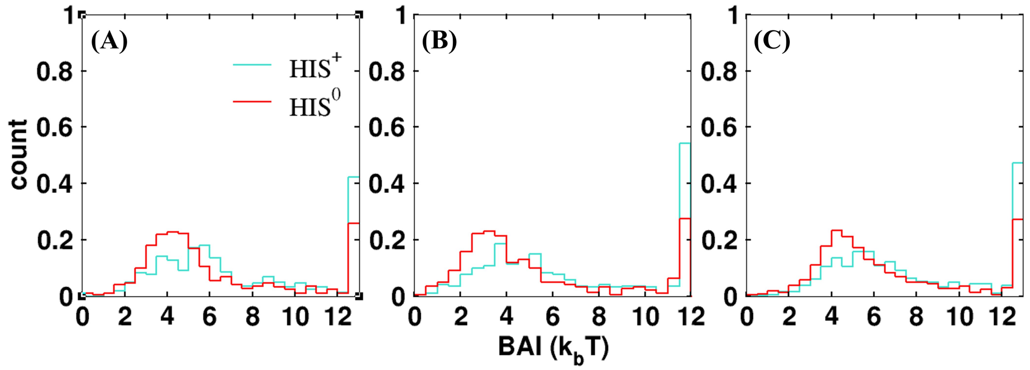

Figure S8: **The BAI distribution for (A) Fc, (B) Fab and (C) COE3 for the complete range of BAI values. There are residues for which we did not identify any contacts with histidine during the analysis of our trajectories. To include the population of these non-contact residues in the probability distribution, we allocate a BAI value equal to INT(BAI_{max}) + 1, where BAI_{max} is the maximum of all BAI values corresponding to residues with at least 1 contact with histidines and "INT" represents the integer of BAI_{max}. These non-contact residues are represented in a single bar centered at the largest BAI for each system (13 for A and C, and 12 for B).**

Table S1: **Amino acid hydrophobicity (from the Black and Mould Scale normalized such that Gly has a hydrophobicity of 0), and $\mathbf{SAA}_{exposed}$ of the sidechain for different central residues. The SAA of residue X was computed in pure water using the *Ala−X−Ala* trimer as described in the next section.**

**Amino acid**	**Hydrophobicity**	$\mathbf{SAA}_{exposed}$ (nm^2)
Ala	0.115	0.8819
Arg	-0.501	2.5819
Asn	-0.265	1.5714
Asp	-0.473	1.3939
Cys	0.179	1.2328
Gln	-0.250	1.9099
Glu	-0.458	1.7785
Gly	0.000	0.4446
His	-0.336	1.9039
Bare His	-0.336	4.225
Ile	0.442	1.8721
Leu	0.442	1.8722
Lys	-0.218	2.2582
Met	0.237	1.9684
Phe	0.499	2.2303
Pro	0.210	1.1826
Ser	-0.142	1.1436
Thr	-0.051	1.4026
Trp	0.377	2.6130
Tyr	0.379	2.3409
Val	0.324	1.5479

SAP calculation procedure

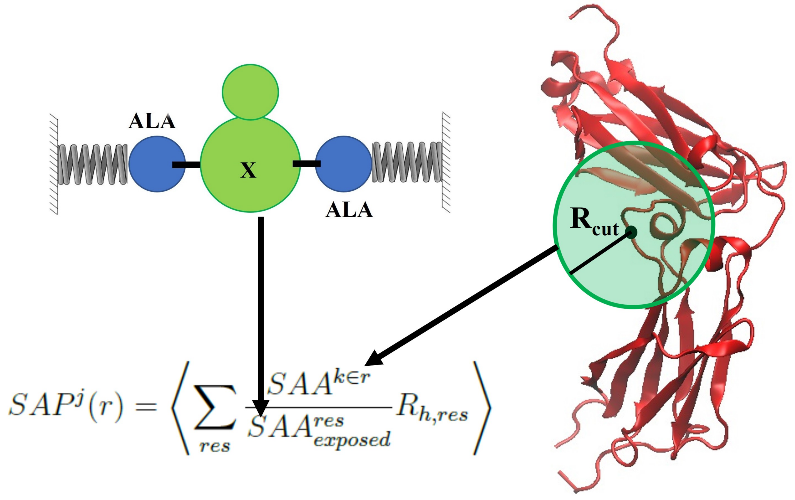

Figure S9: **Schematic showing SAP calculation procedure.**

We used the following methodology to calculate the SAP:

1. First, we generated a neighbour-list for each atom j of the Fab/Fc fragment containing atoms lying within a cut-off distance R_{cut} = 0.5 nm (see Figure S9).

2. The atoms in the neighbour-list were grouped based on the residues they belonged to (res) and the SAA was calculated using a probe radius of 0.2 nm. A probe radius of 0.2 nm is larger than the usual value of 0.14 nm, and it was used for consistency with the BSAP calculations. For BSAP this probe radius allows the inclusion, in the calculations, of charged histidines that form salt-bridges with the GLU and ASP residues of the protein.

3. The SAA of fully exposed residues of type res was calculated by performing a 50 ns long simulation of a Ala–res–Ala tripeptide in pure water, with the CA atoms of the two Alanines restrained, and averaging the SAA of res over the frames of the 50 ns long trajectory.

Figure S6 provides an graphical definition of the variables calculated showing how they enter in the equations to calculate the SAP index.

4. Steps 1-3 were performed for each atom in each frame of the Fab/Fc/COE3 trajectory. The average SAP for each atom was calculated by averaging over each frame of the trajectory.

5. The SAP per residue was calculated by averaging over the SAP's of all the atoms belonging to that residue.

Difference between BSAP and SAP indices

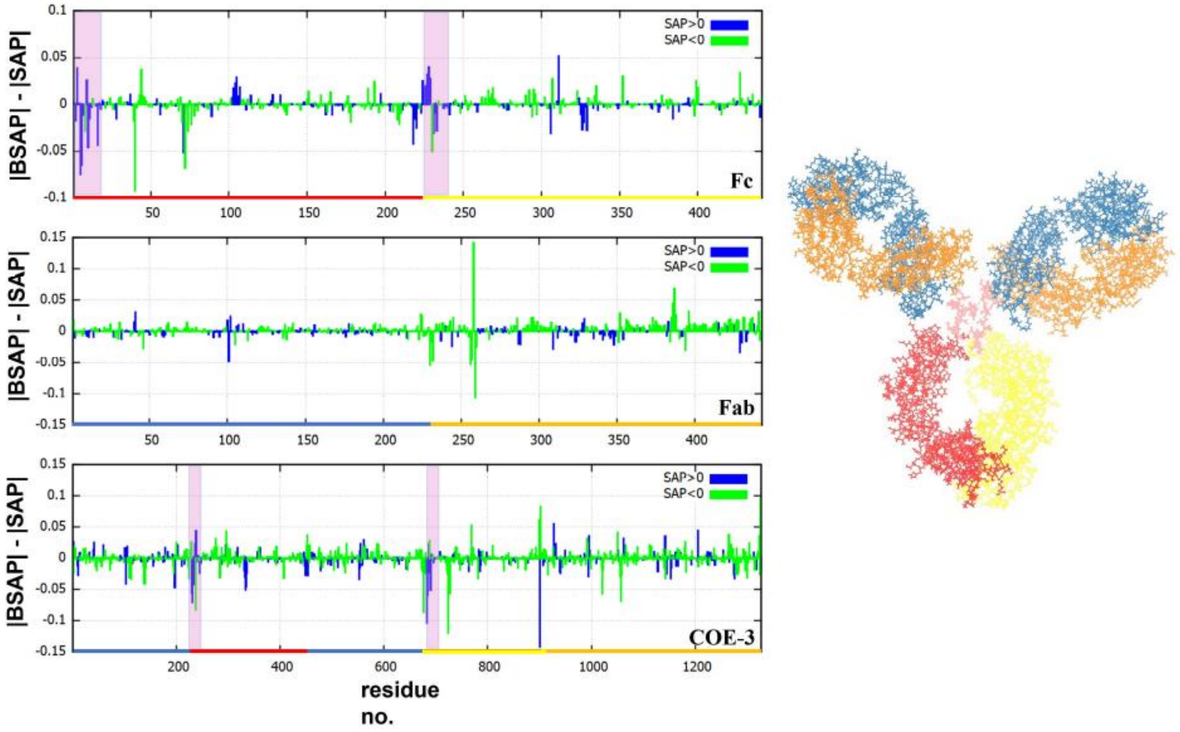

Figure S10: **The difference between the absolute values of BSAP and SAP ($|BSAP| - |SAP|$) for each amino acid residue in the Fc and Fab fragments and COE3. The colours on the x-axis correspond to different regions in the protein sequence (see snapshot of the COE3 structure highlighting the different protein regions). The residues belonging to the hinge region are highlighted by mauve vertical stripes. The $|BSAP| - |SAP|$ for residues with SAP $>$ 0 (hydrophobic) and SAP $<$ 0 (hydrophilic) are shown in blue and green respectively. The absolute values ensure that a reduction in either hydrophobicity or hydrophilicity of a residue in the presence of buffer has a negative sign while an increase has a positive sign.**

Stretched exponential fits for the survival functions

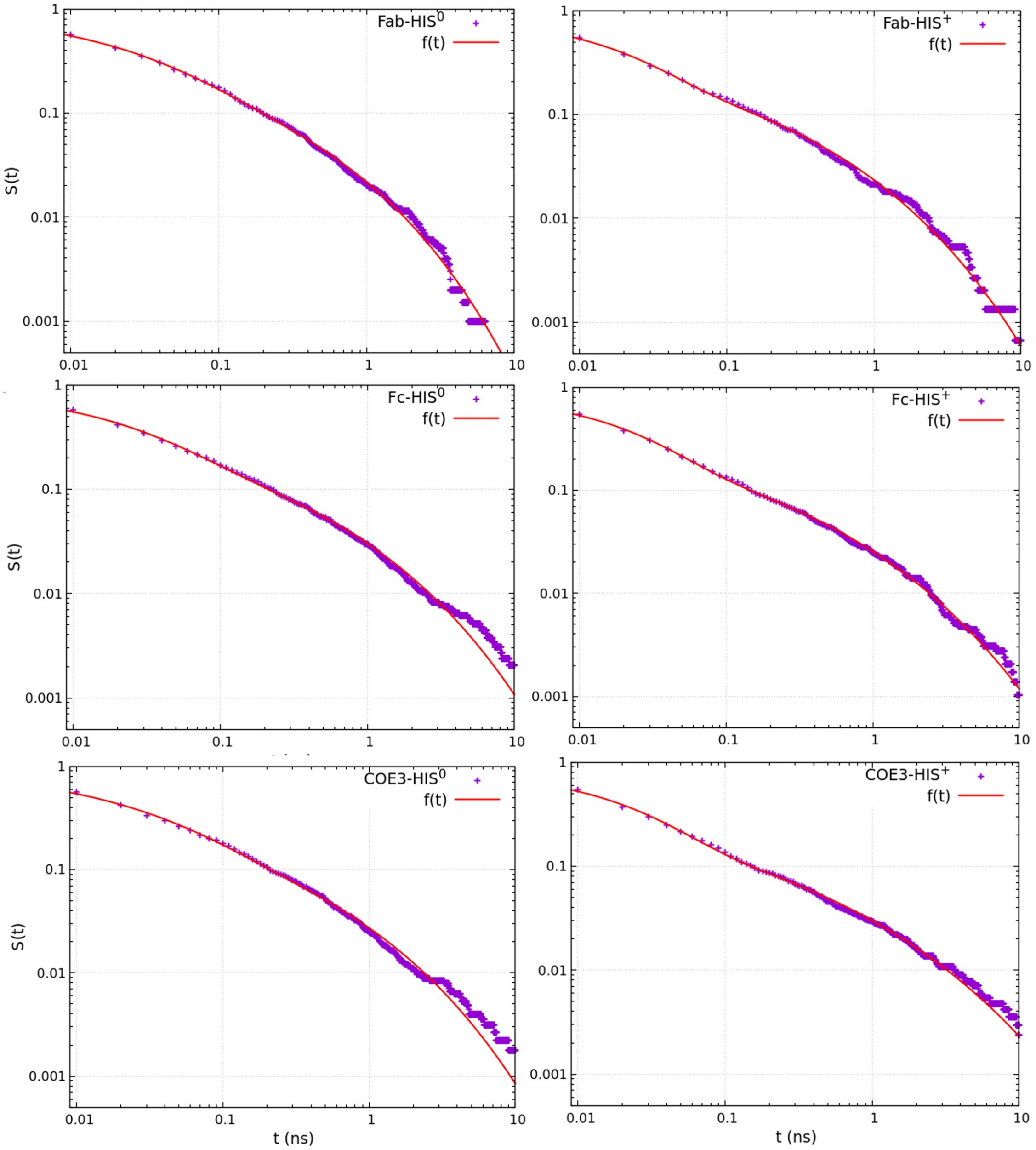

Figure S11: **The survival probabilities and the fitting function: f(t) = $0.5e^{-(k_1 t)^{\mu_1}} + 0.5e^{-(k_2 t)^{\mu_2}}$. A value of 0.5 was used for the constants as a free assignment of values to A led to unusually different values for the two rate constants for some of the systems.**

Time dependence of histidine-binding regions on the Fab/Fc surface

To identify the regions on the protein surface that interact more favourably with histidine, we constructed a time-dependent contact map by superimposing on the protein structure the atoms of buffer histidine that lie within 0.4 nm of the protein surface. We monitored the adsorption at 10 ns intervals along one of the three MD trajectories (see Figures S12 and S13). The contact maps show that histidine binds preferentially to regions containing positively charged and hydrophobic residues (indicated by red circles). Histidine features a more homogeneous distribution at the surface of the the Fc fragment (c.f Figures S4 adn S5). As discussed in the main text, the histidine-protein contacts are expected to influence the inter-fragment (Fab-Fab, Fc-Fc and Fab-Fc) interaction and the aggregation behavior of the proteins.

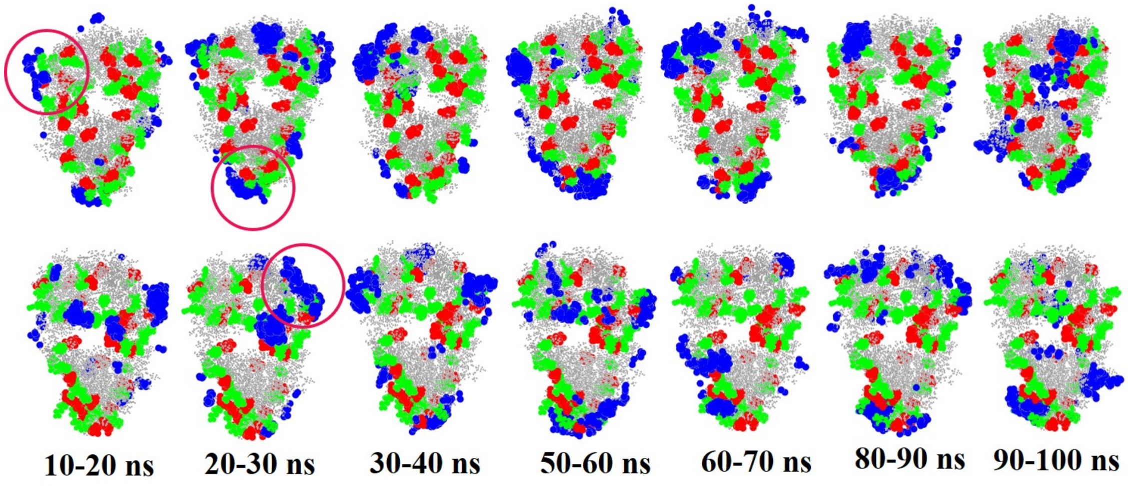

Figure S12: **Contact maps showing the binding regions of histidine on the surface of the Fab domain, for different time intervals. Atoms belonging to the buffer histidine lying within 0.4 nm of the Fab surface are shown in blue. The Fab conformations shown in the figure have been obtained by averaging over frames in the corresponding time interval. The top panels show a front view of Fab and the bottom panels the corresponding back-view. The positively (LYS, ARG) and negatively (GLU, ASP) charged residues on the surface of Fab are highlighted in green and red, respectively.**

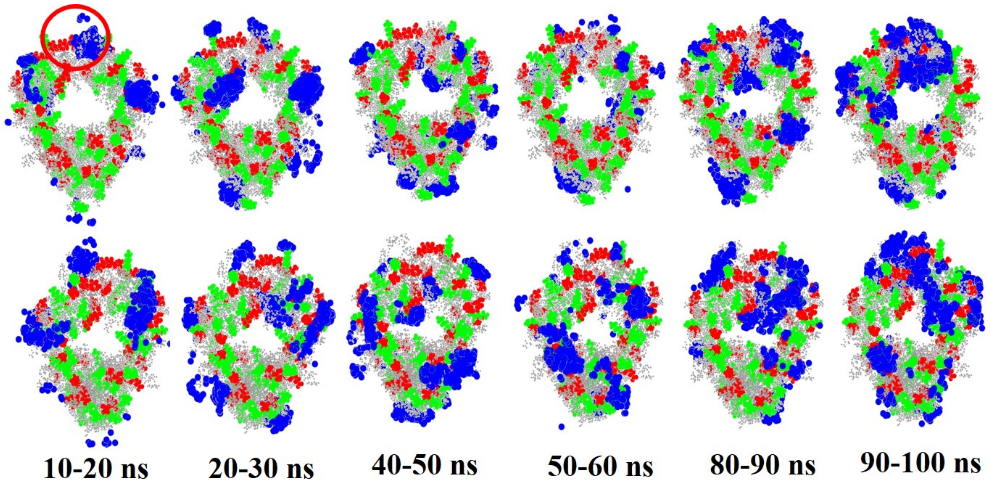

Figure S13: **Same as Figure S7 for the Fc domain.**

Effect of HIS-Protein interaction on histidine diffusion

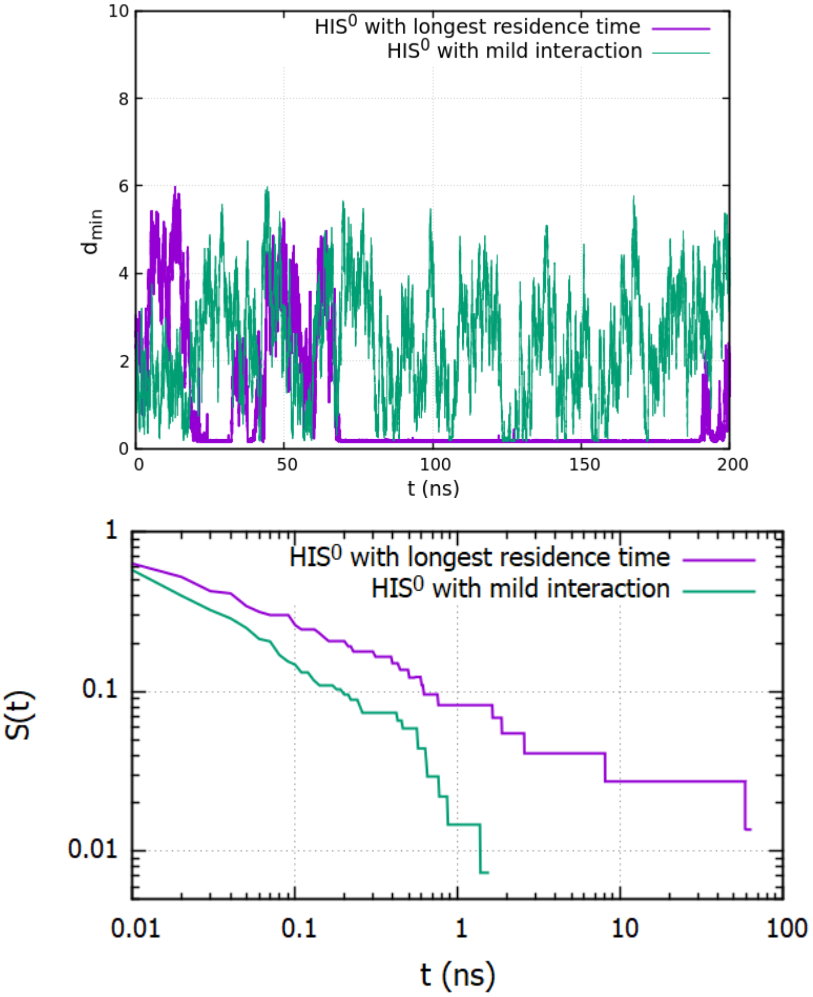

Figure S14: **The minimum distance from the protein surface, HIS^0 molecules, one with the largest residence time on the protein surface and the other the lowest, as a function of time. Difference in survival functions of the interaction of two HIS^0 molecules with the protein surface.**
